# Scalable Electrochemical Concrete Fabrication and Closed‐Loop Recycling

**DOI:** 10.1002/advs.202523927

**Published:** 2026-02-25

**Authors:** Hanxiong Lyu, Zhenlin Li, Yang Liu, Lu Zhu, Lucen Hao, Mingxin Shi, Shipeng Zhang, Chi Sun Poon

**Affiliations:** ^1^ Department of Civil and Environmental Engineering and Research Centre for Resources Engineering Towards Carbon Neutrality The Hong Kong Polytechnic University Kowloon Hong Kong

**Keywords:** carbon‐neutral concrete, circular construction, concrete waste valorisation, electrochemical upcycling, sustainability

## Abstract

Concrete builds the modern world, yet its production remains fundamentally unsustainable. Over 30 billion tonnes of concrete are produced annually, fuelling a one‐way flow of resource extraction and accumulation of CO_2_ emissions and inert demolition waste. Current recycling practices predominantly downcycle waste into low‐grade applications, limiting environmental benefits and stalling progress toward a circular economy. Here, an electrochemical‐driven recycling (ER) strategy is proposed that upcycles concrete waste into high‐value resources, including pristine aggregates and zero‐carbon clinker precursors. The ER process selectively decomposes hydrated cement phases, demonstrating the chemical reversibility of hydration products and enabling regeneration of clinker precursors. The recovered substances are recombined to create reversed Portland Cement and reversed concrete that match conventional counterparts in both composition and performance. Validated at pilot scale (19.2 t/year), the ER strategy achieves 95.4 wt.% circularity and reduces global warming potential from 448.4 to −4.6 kg CO_2_‐eq/m^3^, rendering the concrete carbon‐neutral across its full life cycle. Cost analysis indicates competitiveness with conventional production, offering up to $94.8/m^3^ in net savings under high landfill pricing. By closing both material and carbon loops, this work charts a scalable path toward circular concrete, where demolition no longer marks an end, but a beginning.

## Graphical abstract

The electrochemical‐driven recycling strategy establishes a reversible concrete material flow by extracting Ca, Si, Al, and Fe from concrete waste and regenerating functional feedstocks. These reversed products are reassembled into new concrete, which re‐enters service and ultimately returns to waste, enabling a closed‐loop, waste‐origin, and low‐carbon concrete cycle.

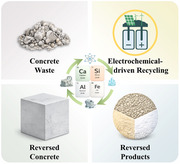

## 1 Introduction

Concrete quite literally holds modern civilization together, yet it anchors global infrastructure to one of the most carbon‐intensive material systems in use today. As the second most consumed material after water, concrete is indispensable and currently lacks scalable alternatives. However, this indispensability comes at an immense environmental toll: over 30 billion tonnes of natural aggregates and 5 billion tonnes of mineral resources are extracted annually to sustain its inherently linear, single‐use production model [1, 2, 3], while the cement industry alone accounts for ∼8% of total anthropogenic CO_2_ emissions globally, emitting 3 billion tonnes of CO_2_ in 2022 [4]. Despite decades of technical progress, including the integration of supplementary cementitious materials (SCMs), the use of recycled aggregates, and the development of carbon capture [5, 6, 7], no strategy to date has fundamentally resolved the dual challenges of material circularity and deep decarbonization in concrete.

Amid these unresolved challenges, an overlooked opportunity lies not in new materials but in old concrete. Generated from the demolition of aging buildings and infrastructure, over 5 billion tonnes of concrete waste are produced annually [8, 9], and this volume is projected to increase in the coming decades, forming one of the largest and most underutilized waste streams on Earth. Although often dismissed as inert debris, this colossal material reservoir harbours untapped regenerative potential. Its two primary components, spent aggregates and hydrated cement paste, contain all the elemental ingredients required to reconstruct high‐performance concrete. Aggregates retain their physicochemical properties and remain reusable even after decades of service. More importantly, the cement paste, though irreversibly hydrated, is rich in calcium, silica, alumina, and iron, which are the core elements needed to produce new cement. Unlike the carbon‐intensive limestone feedstock, the calcium‐bearing phases (e.g., portlandite and calcium silicate hydrates) are inherently decarbonized and release no CO_2_ upon reclinkering. In principle, concrete waste contains everything needed to rebuild concrete, without the carbon cost.

Yet this promise remains technically elusive. A persistent barrier lies in the inability to cleanly and selectively separate hydrated cement paste from embedded aggregates. Conventional mechanical separation technologies fall short because they leave residual paste on the surface of spent aggregates, which triggers microcracks and significantly weakens the interfacial transition zone in new concrete, ultimately compromising strength and durability [10]. To address these deficiencies, various surface treatment techniques have been explored, such as carbonation mineralization [11, 12, 13], pozzolanic slurry coating [14, 15, 16], and polymer impregnation [17, 18]. However, these methods address concrete waste in a component‐specific manner, targeting either aggregate recovery or surface modification, and offer only partial solutions. They often lead to the irreversible loss of cement paste and suffer from high variability in recovery rates, intensive resource demands, and limited economic viability [19]. Further discussions of current concrete waste recycling strategies are provided in Appendix SA.

Even in cases where partial separation of cement paste is achieved, additional fundamental challenges remain. The recovery and regeneration of hydrated cement paste, the most carbon‐intensive and chemically valuable component of concrete, are rarely addressed in a manner compatible with high‐performance cement production. Efforts to reconstitute cement clinker from waste‐derived materials frequently yield altered mineral phases or impaired reactivity due to impurities such as chlorides and sulphates [20], producing substandard binders. Even minor compositional deviations or trace contamination can lead to the outright rejection of Ordinary Portland Cement OPC) under today's stringent performance standards. As a result, most of demolished concrete is still landfilled or downcycled into low‐value applications like road subbase [21, 22]. The vision of circular, carbon‐neutral concrete remains frustratingly out of reach.

Against these limitations, electrochemical technologies have recently emerged as a promising route for cement decarbonization and recycling. Recent studies have demonstrated that electrochemical reactors can decarbonate carbonate feedstocks via in situ pH gradients to recover Ca(OH)_2_ as clinker precursors [23, 24, 25], and extend these concepts to cementitious waste streams through electrolytic extraction of Ca‐ and Si‐bearing phases [26, 27]. However, these advances remain largely cement‐centric and do not yet address the coupled recovery of hydrated cement paste and aggregates required for concrete circularity.

**FIGURE 1:**
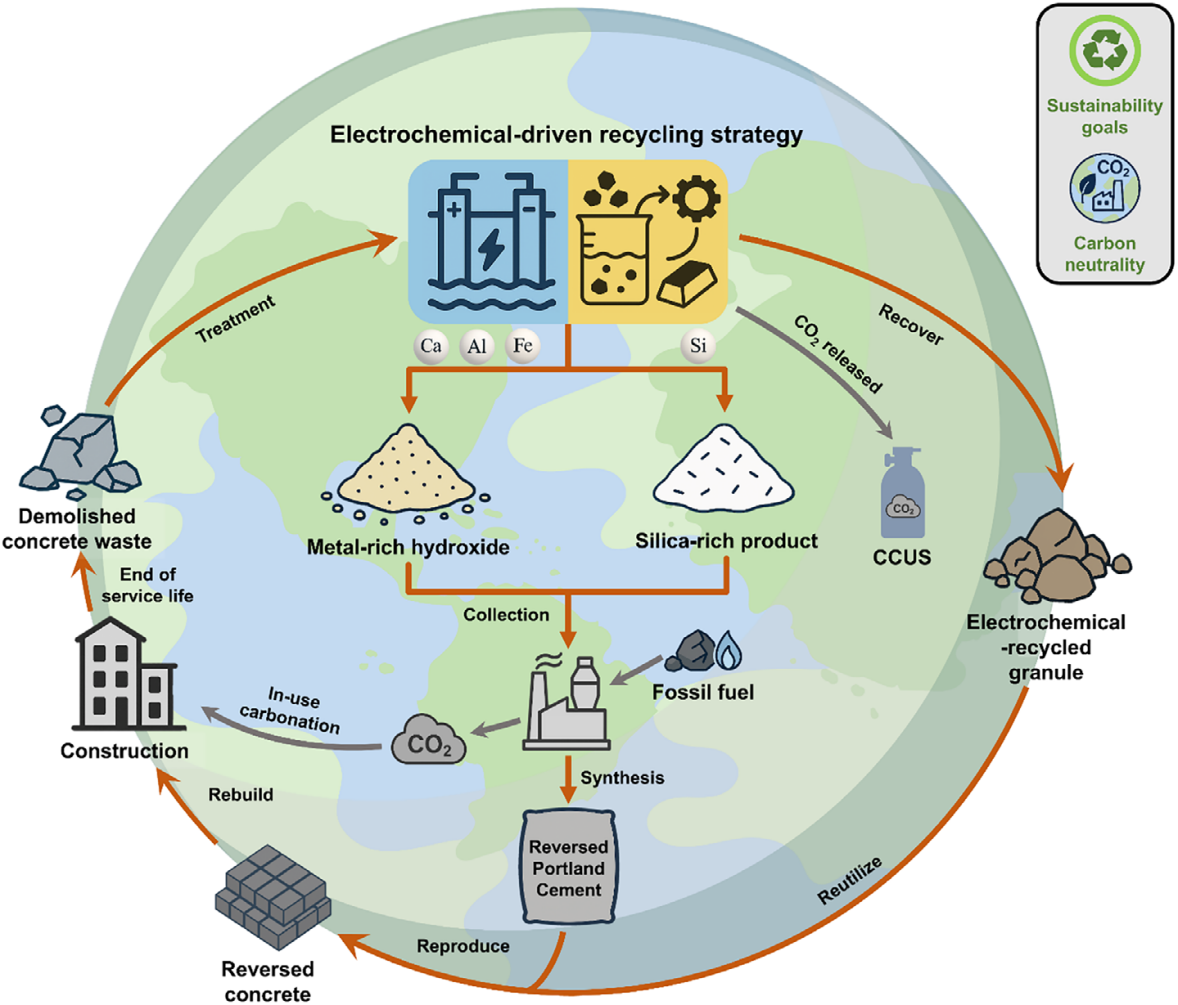
Schematic illustration of the sustainable construction industry. The circular process encompasses several key stages, beginning with the electrochemical treatment of concrete waste and progressing through clinker precursor resynthesis, recovery of sand and aggregate, cement resurrection, reversed concrete manufacture, and construction rebuild. By conducting the electrochemical‐driven recycling strategy, hydroxides and silica precipitations recycled from concrete waste serve as ideal zero‐carbon precursors for cement making. Sand and aggregate recovered via the electrochemical processes fully substitute natural aggregate in reversed concrete production. In parallel, the CO_2_ released from carbonate decomposition during the ER process can be captured for utilization and storage (CCUS). For the CO_2_ emitted from fossil fuel combustion during cement making, it will be reabsorbed through the in‐use carbonation of reversed concrete over its service life. By adopting the proposed strategy, a closed‐loop and carbon‐neutral concrete material cycle is established.

Here, a scalable electrochemical‐driven recycling (ER) strategy is presented that redefines concrete waste from inert debris into an upcycled feedstock for circular and carbon‐neutral concrete production (Figure 1). Through electrochemical processing, concrete waste is decomposed into calcium‐rich metal hydroxides, high‐purity silica, and pristine aggregates. The regenerated constituents are subsequently recombined to enable the synthesis of reversed Portland Cement (RPC) and reversed concrete (RC), establishing a closed material loop between concrete waste and new construction materials. By aligning selective chemical recovery with existing cement manufacturing routes, the proposed ER strategy provides a general framework for upcycling concrete waste into high‐value cement and concrete products and offers a foundation for scalable and circular utilization of demolition‐derived resources.

## 2 Results and Discussion

### 2.1 Electrochemical‐Driven Recycling Strategy

**FIGURE 2:**
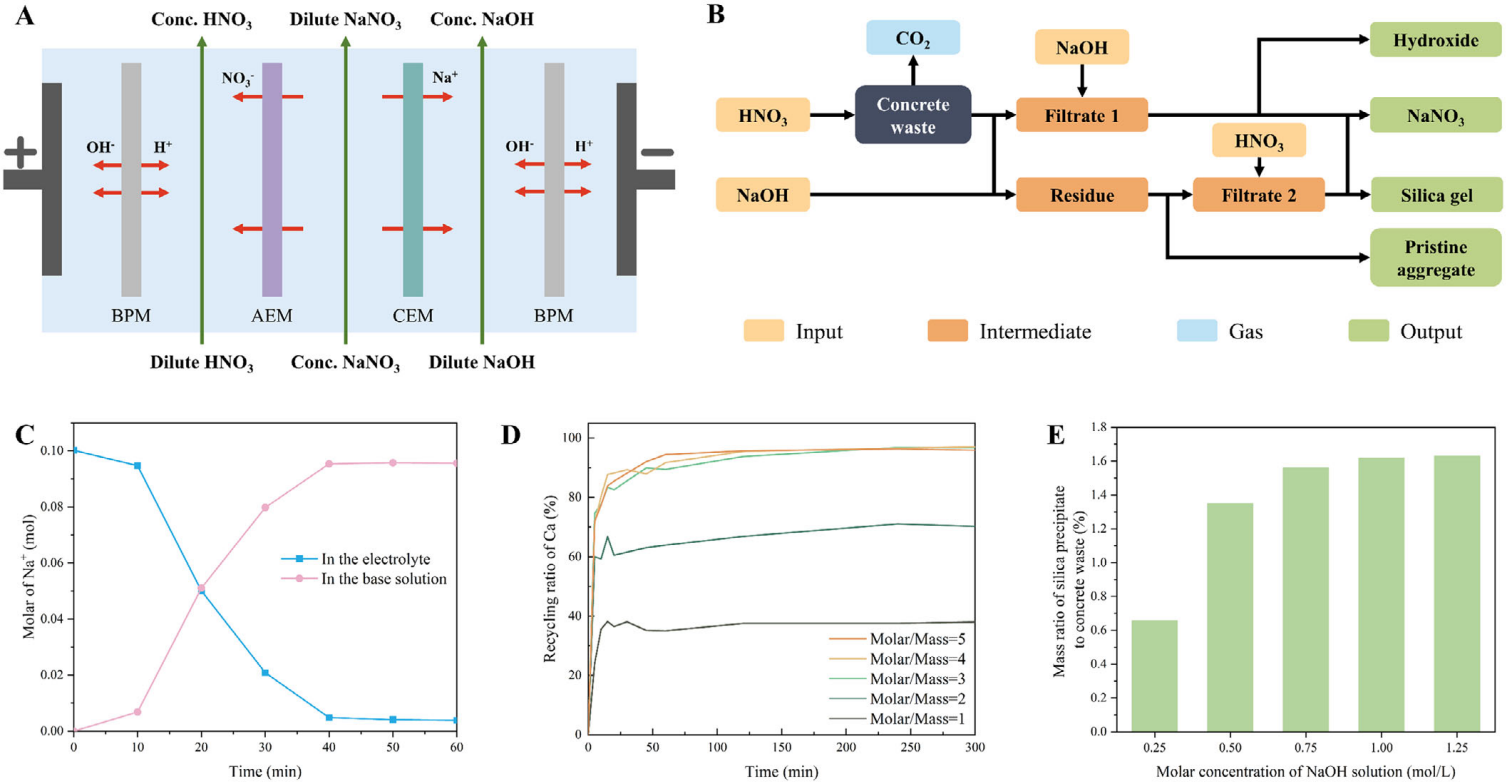
Schematic for an electrochemical‐driven recycling strategy on concrete waste. (A) BPED module; (B) Concrete waste recycling route; (C) Na^+^ ions migration over time in the electrolyte and the base solution; (D) The time‐dependent evolution of recycling ratio of Ca under varying Molar/Mass. (E) Mass ratio of silica precipitate to the concrete waste mass under different NaOH molar concentrations.

The proposed ER strategy enables the targeted dissolution and selective recovery of valuable components from concrete waste. The integrated process comprises two functional modules: an electrochemical module for in situ acid/base generation (Figure 2A) and a treatment module for sequential chemical extraction and material recovery (Figure 2B).

In the electrochemical module, a bipolar electrodialysis (BPED) system was employed to facilitate the generation of HNO_3_ and NaOH from an initial NaNO_3_ solution. Under the applied electric field, Na^+^ ions migrate through the cation exchange membrane (CEM) into the base chamber, while NO_3_^−^ ions migrate through the anion exchange membranes (AEM) into the acid chamber. Simultaneously, water dissociation at the interface of the bipolar membranes (BPM) generates H^+^ and OH^−^ ions, which enter the acid and base chambers, respectively. Driven by the requirement of electroneutrality, each Na^+^ ion transferred must be accompanied by a corresponding NO_3_^−^ ion to the acid chamber, enabling the in situ, paired generation of HNO_3_ and NaOH. As evidenced by the continuous decline in the Na^+^ concentration of the feed solution (Figure 2C), the system achieved effective cation transport and alkali production. Notably, after 60 min of operation, the majority of Na^+^ ions were transferred to the base chamber, thereby validating the BPED system's efficacy in acid/base generation from a single salt precursor.

The treatment module leveraged the generated HNO_3_ and NaOH for component‐specific dissolution and recovery. In Step I, the HNO_3_ solution was used to dissolve the metal oxide present in alkaline hydrates from the demolished concrete waste (Figure S1 and Table S1), releasing Ca^2+^, Al^3+^, Fe^3+^, and Mg^2+^. Simultaneously, calcite formed from weathering carbonation during the service life of concrete decomposes (Figure S1), yielding gaseous CO_2_ and additionally dissolved Ca^2+^. The extent of metal leaching was highly dependent on the initial ratios of acid molarity to solid mass (Molar/Mass), which in turn governs the final pH of the solution and thus the recycling efficiency. The Ca^2+^ ion serves as a key indicator of dissolution progress. As shown in Figure 2D, low Molar/Mass conditions (1 and 2) yielded a limited recycling ratio of ∼40% and ∼70%, respectively, due to final pH values (up to ∼10.4). When Molar/Mass was ≥3, the final pH was below 3.0, enabling nearly complete leaching with ∼95% recycling. Increasing Molar/Mass accelerated the rise in recycling efficiency but did not further improve the maximum recovery ratio. Therefore, the optimal condition was identified as a Molar/Mass of 3 with a 3 h treatment time. The resulting metal‐rich leachate was then treated with NaOH to raise the pH above 12.5, inducing the precipitation of metal hydroxides at 25°C (Figure S2A).

Moving to Step II, the acid‐leached residue was subjected to alkaline treatment using NaOH solution at a slightly elevated temperature of 90°C. These conditions facilitate the reaction between OH^−^ and the silica‐bearing phases [28], forming soluble silicate. The resulting silicate‐rich solution was subsequently neutralized with HNO_3_, triggering the precipitation of amorphous silica in the form of a white, flocculent solid at pH 7.0 (Figure S2B). As shown in Figure 2E, silica yield increased with NaOH molar concentration but plateaued beyond 1.0 mol/L. A higher concentration of 1.25 mol/L only slightly improved recovery, indicating diminishing returns. Thus, 1.0 mol/L NaOH was selected as the optimal condition for silicate dissolution, balancing chemical efficiency and material yield.

The remaining solid residue was collected as electrochemical‐recycled granules, considered as pristine aggregate (Figure S3). All recycled granules have no portlandite, calcium carbonate phase, and other cementitious phases (Figure S4A), and there is no derivative thermogravimetric peak below 900°C (Figure S4B). Therefore, the ER strategy promises that no hydrated cement paste remains on the recovered granules, a potential alternative natural aggregate.

Notably, the regeneration of NaNO_3_ occurs naturally during the neutralization process. Since Na^+^ and NO_3_^−^ ions do not precipitate or bind with extracted species, they remain intact in solution and can be fully recovered for reuse, enabling a closed‐loop reagent cycle. Theoretically, electricity is the sole net input to the system, driving the conversion of water and NaNO_3_ into HNO_3_ and NaOH, which are ultimately returned to their original state via neutralization. Furthermore, the released CO_2_ gas in Step I can be effectively captured and valorised, such as by injecting it into the calcium‐rich solution for mineral carbonation (Figure S5).

Taken together, this ER strategy establishes a unified, electricity‐enabled route to effectively convert concrete waste into metal hydroxides, silica, and electrochemical‐recycled granules. All of them serve as essential feedstocks for RPC making and ultimately enable the manufacturing of RC with high circularity and a low carbon footprint.

### 2.2 Material Properties of Hydroxide and Silica Precipitations

**FIGURE 3:**
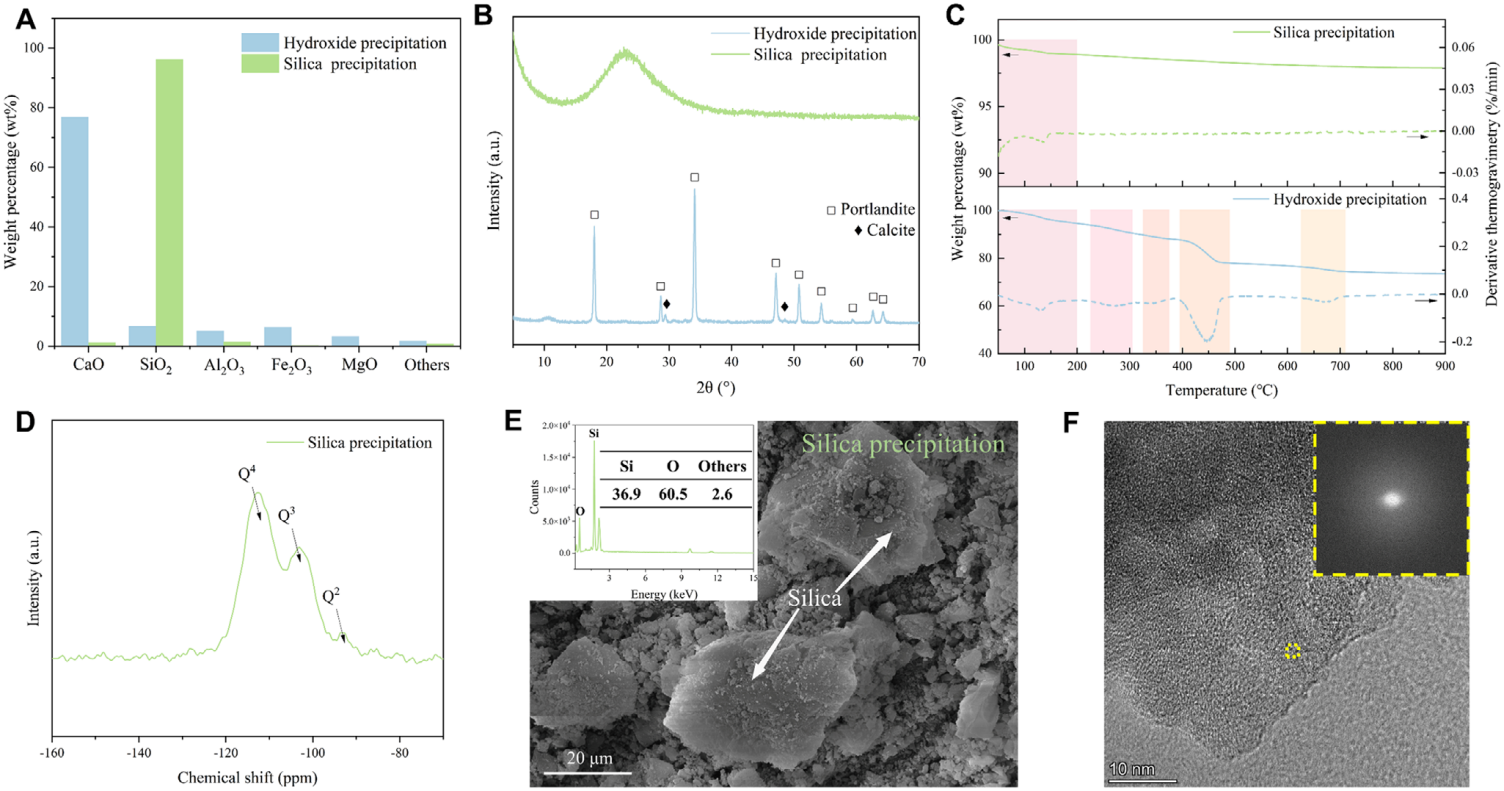
Characterizations of hydroxide precipitation and silica precipitation. (A) Oxide components examined by X‐ray fluorescence. (B) X‐ray diffraction patterns. (C) Thermogravimetric analysis with derivative thermogravimetry. (D) ^29^Si Nuclear magnetic resonance result of silica precipitation. (E) Scanning electron microscope of silica precipitation with energy‐dispersive X‐ray analysis. (F) Transmission electron microscope of silica precipitation with Fast Fourier Transform analysis.

Analytical characterizations of recovered substances synthesized under the optimal ER conditions reveal distinct compositional and structural features. The milky white hydroxide precipitation is primarily composed of CaO (76.9%), followed by SiO_2_ (6.69%), Fe_2_O_3_ (6.32%), Al_2_O_3_ (5.09%), and MgO (3.27%) (Figure 3A; Table S2). This minor amount of SiO_2_ is likely sourced from the marginal dissolution of silica in minerals like albite or silica gel [29, 30]. Composition examination results (Figure 3B,C) identify portlandite as the major component in hydroxide precipitation. The presence of aluminium and iron gels is confirmed by their thermal decomposition events of 225°C–305°C and 325°C–375°C [31, 32] respectively. These results illustrate the effective recovery of metal elements from the residual pastes into valuable hydroxides through the ER strategy. A trace amount of calcite (decomposed between 625°C–710°C) is detected, attributed to the inevitable weathering carbonation during sample preparation.

Silica precipitation is recovered as a white powder, mainly consisting of 96.3 wt.% SiO_2_ in an amorphous state with Q^3^ and Q^4^ silicate polymorphs (Figure 3A, B, and D; Table S2). A minor Q^2^ peak suggests the presence of calcium silicate hydrate (Figure 3D), which is also confirmed by the derivative thermogravimetric peak of around 137°C (Figure 3C), indicating the bound water within the silicate structure. Morphological characteristics further confirm its rich amorphous SiO_2_ nature (Figure 3E,F).

### 2.3 Reversing the Recycled Substances

The viability of utilizing recycled substances in cement making and concrete manufacturing is verified.

**FIGURE 4:**
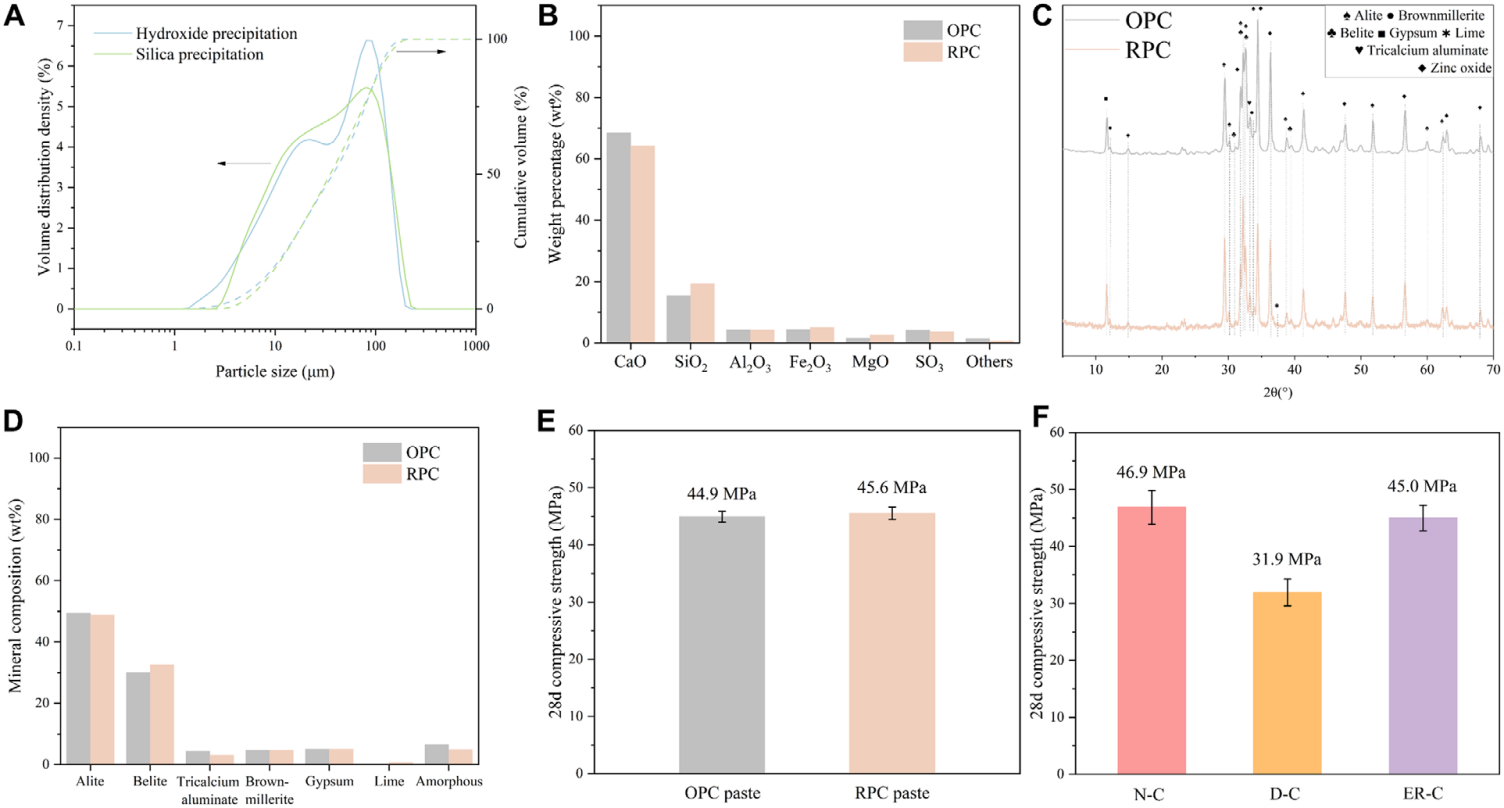
Property and performance characterizations. (A) Particle size distribution of ground hydroxide precipitation and silica precipitation. (B) Chemical oxide composition of RPC and OPC. (C) X‐ray diffraction patterns of RPC and OPC. (D) Mineral phase composition of RPC and OPC. (E) 28d compressive strength of the RPC and OPC paste under normal hydration curing. (F) 28d compressive strength of concrete fabricated with natural resources (N‐C), demolished concrete waste (D‐C), and electrochemical‐recycled granules (ER‐C).

Elementally, the hydroxide precipitate is rich in Ca, while the silica precipitate predominantly contains Si, conveniently providing the major components for Portland clinker production. Therefore, the hydroxide and silica precipitations are repurposed as the exclusive clinker precursors for manufacturing reversed Portland clinker (r‐Pcl). Due to physical agglomeration during drying, a slight grinding was conducted to transform the material into micrometre‐scale powder (Figure 4A; Table S3). Unlike the energy‐intensive grinding used in traditional cement production, this process benefits from reduced energy consumption.

Referring to traditional OPC production, the feedstock is designed based on lime saturation factor (LSF: 99.48), silica modulus (SM: 2.04), and aluminium ratio (AR: 0.84) (Appendix SB). The resulting RPC, composed of 95wt.% r‐Pcl and 5 wt.% gypsum (added to the pulverized r‐Pcl to control the setting behavior of the aluminium‐bearing phase), exhibits an oxide component closely matching that of the OPC reference (Figure 4B; Table S4). Quantitative phase analysis further indicates that the RPC consists of 48.8 wt.% alite, 32.7 wt.% belite, and minor phases of 3.1 wt.% tricalcium aluminate, 4.7 wt.% brownmillerite, 5.0 wt.% gypsum, and 4.9 wt.% amorphous phase (Figure 4C,D; Table S5), comparable to that of OPC. A 0.7 wt.% free lime is detected, well within the generally advisable limit of 1.5% [33], indicating the good quality of this clinker in terms of soundness and expansion control [34]. The content of alite and belite, two primary phases in cement, falls within the typical composition range specified for API and ASTM cement standards [35], affirming the suitability of these recovered materials as sole feedstock in cement production.

The obtained RPC and OPC powders both appear grey (Figure S6) due to the presence of iron‐containing phases like brownmillerite within the cement [36, 37], and exhibit similar particle size distribution (Figure S7 and Table S6). Mechanical performance further supports the viability of RPC. At 28 days, the RPC paste achieves a compressive strength of 45.6 MPa, comparable to the OPC reference (44.9 MPa, Figure 4E). These findings demonstrate that RPC, synthesized from electrochemically recovered hydroxide and silicate precipitates, is not only a functional substitute for OPC but also a scalable route toward closed‐loop, low‐carbon cement production.

In addition to cementitious precursors, the ER process yields granular products that are sieved into powder (ER‐P, 0‐0.3 mm), sand (ER‐S, 0.3–2.36 mm), and aggregate (ER‐A, 2.36–10 mm) (Figure S3). The first visual impression is that the ER process effectively removed residual cement paste from the spent aggregate, resulting in a cleaner surface for the ER batch compared to untreated ones. Since the old paste has a higher water absorption capacity than the gravel, its removal is also reflected in the reduced water absorption ratio, aligning the electrochemical‐recycled granules with that of standard sand and natural aggregates (Table S7). The feasibility of their practical application is verified by concrete strength evaluation, where OPC was used as the binding material to avoid interruption (mix design in Table S8). The concrete (ER‐C) fabricated with ER‐S and ER‐A achieves a 28d compressive strength of 45.0 MPa, rivalling that of natural resources‐based concrete (N‐C, Figure 4F). Conversely, in the scenario where demolished concrete waste was utilized directly as the aggregate of the concrete (D‐C), a 32.0% reduction in strength is observed. The primary cause of strength loss in D‐C is the presence of old paste and the associated weak/multiple interfacial transition zone [38]. The strength results of concrete with different aggregates further verify the significance of the ER process that restores aggregate quality.

ER‐P, the finest portion in electrochemical‐recycled granules, primarily comprises quartz in mineral phases or as SiO_2_ in oxide form, similar to ER‐S and ER‐A (Figure S4A and Table S9). These attributes make ER‐P comparable to granite powder [39], typically generated as the by‐product of granite mining. Due to the requirement for an aggregate size distribution matching that of N‐C, ER‐P was not included in the ER‐C fabrication (Table S8). However, inspired by the use of granite powder, ER‐P can be reutilized in concrete fabrication, such as filler materials and SCMs [39, 40]. The fine particle size of ER‐P could provide nucleation sites for cement hydration, enhancing strength development [41].

To assess the efficiency of the ER strategy in converting concrete waste into valuable substances, a parameter, the circularity index, is calculated by comparing the total mass of the reversed substances (r‐Pcl and electrochemical‐recycled granules) with the mass of input concrete waste heated to 900°C. The heating step targets to obtain net cement and aggregate, removing the effects of bonded water and any mineralized CO_2_, thus allowing for an accurate evaluation of recycling efficiency. Overall, a circularity index of 94.5 wt.% is achieved (Table S10), indicating nearly all input materials are successfully recycled or repurposed. Such a high circularity index highlights the efficiency of this ER approach, advancing sustainability and circular economy principles in the construction industry.

Moreover, the performance of the reversed materials demonstrates that they are viable substitutes for natural resources commonly used in concrete production. This substitution would mitigate the pressure on resource‐intensive extraction, reducing the environmental impacts at the cradle stage. Notably, the traditionally used calcium supplier, limestone, theoretically contains 44 wt.% CO_2_, which is released during the calcination process, accounting for 50%–60% of the total CO_2_ emissions of cement making [42, 43]. In contrast, the calcium‐rich hydroxide synthesized from concrete waste can be considered as a decarbonized material (Figure 3B,C). Its adoption significantly reduces the carbon footprint of the resulting cement.

### 2.4 Pilot‐Scale Validation with Real‐World Concrete Waste

Building on the lab‐scale study of the ER strategy, robustness and scalability under practical conditions are assessed. Unlike the controlled conditions in a lab‐simulated scenario, the real‐world concrete waste is inherently heterogeneous, particularly from a compositional perspective. This variability arises from differences in original mix designs, the presence of diverse mineral admixtures and chemical additives, and long‐term environmental exposure during service life. As a result, batch‐to‐batch fluctuations are common and have long limited the efficiency of conventional recycling practices, where waste is typically repurposed as recycled aggregate with unpredictable performance [44, 45].

In contrast, the ER process is inherently element‐oriented, enabling the selective extraction of key constituents (e.g., Ca, Si) regardless of upstream heterogeneity. To validate the adaptability of this approach under industrially relevant conditions, a pilot‐scale ER system was established with an annual processing capacity of 19.2 tons (Figure S8A). The pilot system integrates three functional modules: (i) electrochemical unit, (ii) product processing (equipped with pH, conductivity monitoring, temperature, flow rate, and filtration controls), and (iii) liquid circulation. The system was operated using real concrete waste sourced from a commercial recycling plant in Hong Kong (HK), representing a typical heterogeneous feedstock. Despite the input variability, the ER process successfully produced calcium‐rich precipitate (hydroxide(HK)) and silica‐rich precipitate (silica(HK)) (Figure S8B and Table S11). Aligning with the laboratory results, the hydroxide(HK) has a primary mineral phase of portlandite, and the silica(HK) possesses a high‐purity silica ratio of 98.6 wt.% with an amorphous nature (Figure S8B–D and Table S11). Importantly, chemical analysis shows that chloride and heavy metal oxides are present only at trace levels in the recovered products, while sulfate contents remain at levels well below those typically introduced during conventional cement production through gypsum addition. These results indicate that, even when processing real‐world concrete waste, common impurities such as chlorides, sulfates, and heavy metals do not show significant accumulation in the recovered solid products.

By exclusively using these two substances (Appendix SB), the r‐Pcl(HK) clinker that closely replicates the mineral composition of OPC is also successfully produced. It primarily comprises alite (49.2 wt.%) and belite (28.1 wt.%), along with minor phases of tricalcium aluminate (8.5 wt.%), brownmillerite (6.3 wt.%), and an amorphous phase (8.0 wt.%) (Figure S8E). The similarity is also reflected in the comparable strength of paste specimens made from this waste‐derived cement (RPC(HK), composed of 95 wt.% r‐Pcl(HK) and 5 wt.% gypsum) and OPC. The RPC(HK) paste develops a 28d compressive strength of 43.3 MPa. Moreover, an RC(HK) is fabricated using RPC(HK) and the electrochemical‐recycled granules(HK) with the mixture proportion displayed in Table S8. The resulting RC(HK) records a 28d strength of 44.7 MPa, which well satisfies the mechanical requirement for normal structural concrete in HK (35 MPa) [46].

According to the weight percentage of each recycled substance (Table S12) and clinker formulation (Appendix SB), the overall circularity index is determined to be 95.4 wt.% (Table S13), which represents an exceptionally high level of resource recovery for real‐world concrete waste. This result underscores the robustness and scalability of the ER strategy, demonstrating that near‐complete material circularity can be achieved even under heterogeneous and industrially relevant conditions.

### 2.5 Environmental and Economic Assessment

The ecological impact of the proposed electrochemical‐driven cement and concrete was evaluated in terms of the global warming potential (GWP100) within the framework of a pilot‐scale study, using a parallel comparison with their conventional counterparts based on natural resources. The assessment covered the relevant life cycle stages, defined as cradle to gate for cement production and cradle to grave for concrete, including production, in‐use, and end‐of‐life disposal. Before LCA, the energy consumption associated with the ER strategy was quantified based on the pilot‐scale study. Electricity is the sole energy input for the ER process, including BPED and auxiliary operations. The electricity demand comprises BPED operation (0.38 kWh/kg concrete waste), water production (0.0167 kWh/kg concrete waste), mixing (0.0228 kWh/kg), heating (0.0194 kWh/kg), precipitation (0.009 kWh/kg), and filtration (0.0228 kWh/kg), resulting in a total electricity consumption of 0.471 kWh/kg concrete waste. The LCA was performed assuming renewable electricity input, representing the envisioned low‐carbon operating scenario of the electrochemical route. This assumption aligns with the projected decline of global grid emission factors by up to 88% over 2022–2050 [47], highlighting the growing scalability and development potential of the ER strategy under future low‐carbon energy systems. Methodology is depicted in Appendix SC.

**FIGURE 5:**
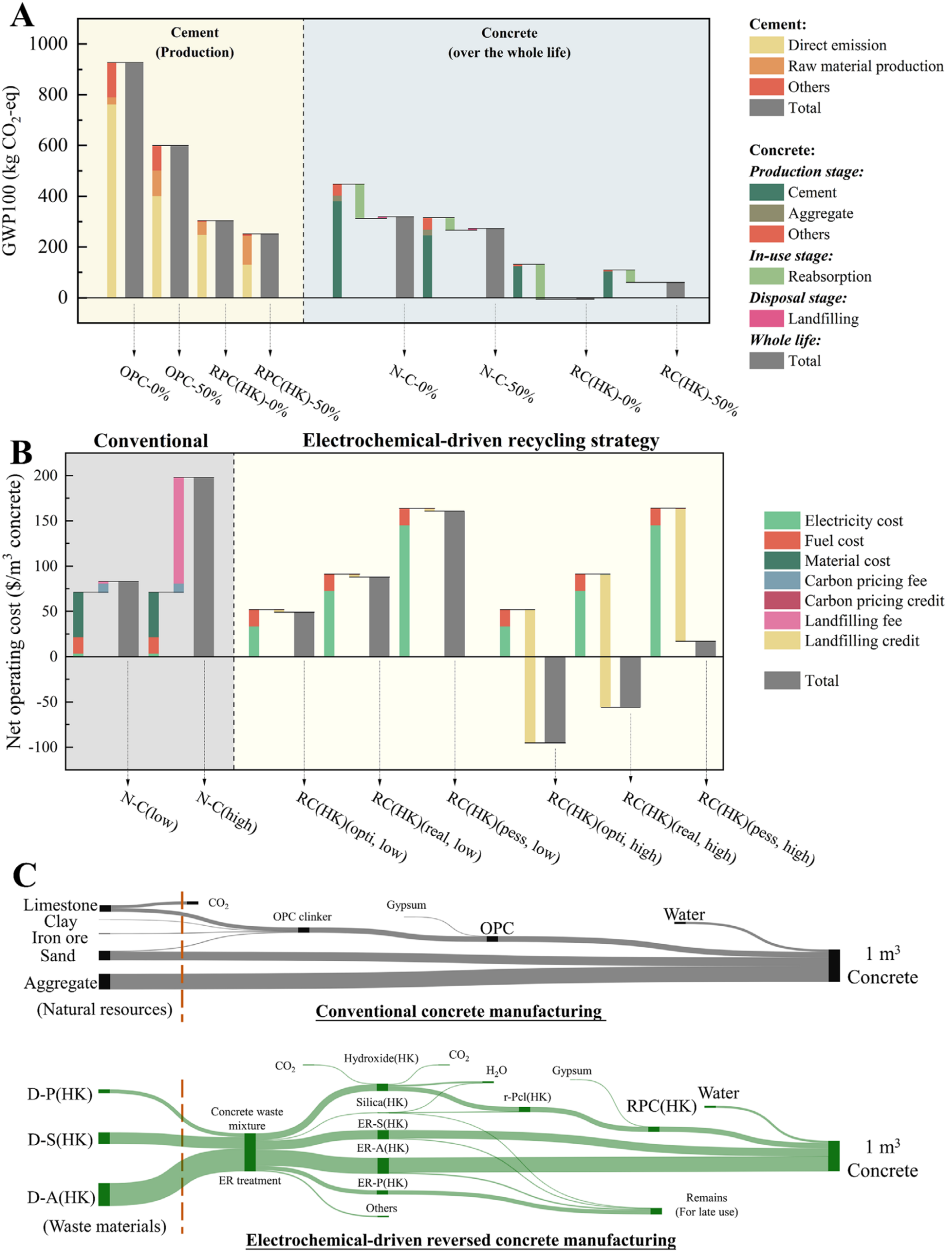
Environmental and economic assessment of the final products. (A) GWP100 of 1 t cement and 1 m^3^ concrete based on natural resources and ER‐derived materials; (B) Net operating cost of 1 m^3^ concrete manufacturing based on conventional and ER strategy; (C) The material flow diagram of the N‐C manufacturing relied on natural resources, and RC(HK) manufacturing relied on recovered materials from concrete waste. (Demolished concrete waste includes powder (D‐P(HK), <0.3 mm), sand (D‐S(HK), 0.3–2.36 mm), and aggregate (D‐A(HK), 2.36–20 mm). The weight ratio of ER‐derived substances corresponds to Table S12. The ‘Remains’ part is composed of the ER‐derived substances that are not used in 1 m^3^ concrete production, which can be used later).

Figure 5A indicates that the cradle to gate GWP100 of conventional OPC manufacturing using fossil fuel (OPC‐0%) is 927.1 kg CO_2_‐eq per ton, comparable to the reported value [48, 49, 50]. Emissions are attributed to in‐kiln limestone decomposition and fuel combustion (761.4 kg CO_2_‐eq), followed by upstream raw material production (29.0 kg CO_2_‐eq), and other electricity‐ and transport‐related activities (136.7 kg CO_2_‐eq). These values underscore the inherently carbon‐intensive nature of traditional cement production, driven by the use of carbonate‐based feedstocks and fossil fuels. To reflect current industry efforts toward decarbonization, the LC^3^ system was introduced, a widely studied and increasingly adopted formulation that substitutes clinker with SCMs [51]. Specifically, the LC^3^ mix comprises 50 wt.% OPC clinker, 30 wt.% calcined clay, 15 wt.% limestone, and 5 wt.% gypsum (OPC‐50%). This configuration lowers the GWP100 to 599.6 kg CO_2_‐eq per ton, marking a 35.3% decrease. The improvement is primarily attributable to clinker reduction, although emissions from the production of calcined clay and limestone partially offset the gains. In contrast, the electrochemically derived RPC demonstrates a more profound carbon reduction. By exclusively using calcium‐rich metal hydroxide and silica as feedstocks, RPC(HK)‐0% achieves a GWP100 of 303.9 kg CO_2_‐eq per ton, representing a 67.2% reduction relative to OPC‐0%. Diverging from the traditional carbon‐intensive limestone as the calcium supplier, the embodied carbon of the ER‐derived calcium hydroxide is zero, and thus its use eliminates process emissions during clinkering, which constitute the dominant source of direct CO_2_ in traditional cement systems. When considering the synergistic effect of SCMs blending and waste‐derived feedstocks, RPC(HK)‐50% achieves the lowest GWP100 overall of 251.8 kg CO_2_‐eq per ton. However, emissions from SCMs processing slightly diminish the marginal benefit compared to pure RPC.

Building upon the cement results, the analysis extended to concrete systems by combining each cement type with either natural or electrochemically recovered aggregates. At the production stage (cradle to gate), the GWP100 of conventional OPC‐based concrete (N‐C‐0%) is 448.4 kg CO_2_‐eq per m^3^, comprising 380.1 kg from cement, 22.4 kg from aggregates, and 45.9 kg from transportation and processing (Figure 5A). Substituting OPC‐0% with OPC‐50% results in a GWP100 reduction to 314.2 kg CO_2_‐eq per m^3^ (N‐C‐50%), consistent with the cement‐level trend. In comparison, the reversed concrete systems RC(HK)‐0% and RC(HK)‐50% achieve a dramatic drop to 129.9 and 108.6 kg CO_2_‐eq per m^3^, respectively, due to the combination of low‐carbon cement and the complete elimination of aggregate‐related emissions.

To ensure a fair whole‐life comparison, the model further incorporated the in‐use stage (gate‐to‐gate), accounting for natural carbonation whereby atmospheric CO_2_ reacts with hydrated cement phases (e.g., portlandite) to form stable carbonates. Previous studies report that concrete made with conventional OPC could reabsorb approximately 30% of carbon emissions over the service life [52, 53, 54]. When half of the OPC is replaced by SCMs, as in OPC‐50%, the uptake is reduced to around 15% due to their lower calcium content [52]. Given the similar mineralogy between OPC and RPC, the same uptake factors were applied to RPC‐based concrete.

At the end‐of‐life stage (gate to grave), different waste management pathways were assumed. N‐C waste was modelled as disposal in inert landfills, with only transport and on‐site handling considered. Secondary carbonation during landfilling was not credited, as it is time‐dependent and excluded to maintain comparability with the RC system. In contrast, RC waste was assumed to be directly reintegrated into the ER process at the end of life, with no separate disposal stage.

After accounting for both in‐use carbonation and end‐of‐life treatment, the adjusted GWP100 for N‐C‐0% and N‐C‐50% remain significantly higher at 319.0 and 272.1 kg CO_2_‐eq per m^3^, respectively. In contrast, the net GWP100 of RC(HK)‐0% drops to −4.6 kg CO_2_‐eq per m^3^, effectively resulting in zero whole‐life embodied carbon emissions, thereby achieving a truly carbon‐neutral concrete solution.

Besides environmental assessment, a comparative evaluation of net operating costs for 1 m^3^ of concrete over the whole life cycle, including production, in‐use, and disposal stages, was also conducted. As shown in Figure 5B, the economic performance of the ER strategy is strongly dependent on landfill disposal fees and electricity prices. For conventional N‐C, the net operating cost ranges from $82.8/m^3^ under a low landfill fee scenario to $197.8/m^3^ under a high landfill fee scenario. Under a high landfill fee condition, the avoided disposal cost in the ER strategy significantly offsets the expenditures, resulting in negative net operating costs as low as $‐94.8/m^3^ under optimistic electricity prices. Even in the pessimistic electricity case, the ER route still outperforms the conventional process ($17.0 vs. $197.8/m^3^). In contrast, under a low landfill fee condition, the cost advantage of the ER strategy becomes marginal and is only maintained when electricity prices are low to optimistic, with costs ranging from $48.9 to $160.7/m^3^ depending on the electricity tier. These results highlight the critical influence of both electricity pricing and landfill policy on the economic feasibility of the ER process. Consequently, while the ER route may not universally outperform conventional concrete in all scenarios, it can become economically competitive or advantageous in regions with high landfill fees and favourable electricity prices, highlighting its strong sensitivity to local market and policy conditions.

Another advantage of RC(HK) manufacturing lies in utilizing waste materials rather than natural resources (Figure 5C), thereby alleviating pressure on natural ecosystems and raw material supply chains. Specifically, the production of 1 m^3^ N‐C requires approximately 2367 kg of natural resources, including limestone, clay, iron ore, and natural aggregates. In contrast, the ER strategy leverages around 2925 kg of concrete waste to manufacture 1 m^3^ RC(HK), representing a complete substitution of raw materials with end‐of‐life construction debris. The material input breakdown is provided in Appendix SD. This approach not only prevents the depletion of primary resources but also diverts a substantial amount of solid waste from landfills or low‐value applications. This transformative shift from linear extraction to closed‐loop regeneration significantly enhances the sustainability profile of concrete production.

## 3 Conclusions

This study demonstrates an ER strategy that redefines concrete as a truly circular and carbon‐neutral material. Through electrochemically driven separation and upcycling of concrete waste, pristine aggregates and CO_2_‐free precursors are regenerated and reconstituted into high‐performance RC. Pilot‐scale validation (19.2 t/year) confirms the technical viability of this closed‐loop process, achieving a circularity index of 95.4 wt.% and a full‐life GWP100 of −4.6 kg CO_2_‐eq per m^3^. Economic evaluation further reveals cost parity or up to $94.8/m^3^ in savings under the high landfill pricing scenario, underscoring the strategy's scalability and practical potential. By achieving high circularity, full carbon neutrality, and economic competitiveness, the ER strategy bridges a long‐standing gap between recycling efficiency and material quality. This represents a critical advancement toward a circular, climate‐positive concrete industry and contributes a practical solution to the global challenge of sustainable infrastructure development.

## 4 Experimental Section

### 4.1 Materials and Methods

In this study, both laboratory‐simulated concrete waste and real‐world concrete waste were adopted to validate the proposed concept.

Laboratory‐simulated concrete waste was generated by crushing concrete made from a typical mix with a water/cement ratio of 0.4 and a fine/total aggregate ratio of 35%. The mix included cement I 52.5 N (Ordinary Portland Cement, OPC), standard sand (0.3–2.36 mm), and natural aggregate (2.36–10 mm). After aging for 2 years, the concrete was crushed and sieved into three categories based on particle size: powder (D‐P, <0.3 mm), sand (D‐S, 0.3–2.36 mm), and aggregate (D‐A, 2.36–10 mm). The bulk oxide components, mineralogical compositions, and physical properties of D‐P, D‐S, and D‐A were determined and are presented in Table S1, Figure S1, and Table S14, respectively. Concrete waste was oven‐dried at 105°C for 24 h to remove the moisture. The mixture containing D‐P, D‐S, and D‐A, in a weight ratio of 1:3:6, was then collected to perform the ER procedures.

Real‐world concrete waste was collected from a local recycling plant in HK and used to perform a pilot‐scale study validating the effectiveness of the electrochemical technology. After collection, the concrete waste was sieved into the particle size categories similar to the laboratory study: <0.3 mm (D‐P(HK)), 0.3–2.36 mm (D‐S(HK)), and 2.36–20 mm (D‐A(HK)). Following drying, the concrete waste mixture was prepared with a weight ratio of 1:3:6 for D‐P(HK), D‐S(HK), and D‐A(HK) to carry out the validation experiments.

NaNO_3_ (AR, purity of 98%), de‐ionized (DI) water, isopropyl alcohol, ethanol, gypsum (AR, purity of 98%), zinc oxide (AR, purity of 98%), and concentrated nitric acid were obtained in the lab without further purification and treatment.

### 4.2 Characterizations

The concentration of metal ions in the solution was detected by ICP‐OES (SpectroBlue). The solution samples were filtered by a 0.22 µm filter membrane, followed by digestion using concentrated nitric acid, dissolution in nitric acid with a concentration of 5%, and filtration through a 0.22 µm filter membrane. The recycling ratio of Ca was determined as the fraction of *m_Ca_* and *M_Ca_*, where *m_Ca_* is the mass of calcium element in the leachate, and *M_Ca_* is the mass of the calcium element in the concrete waste, computed using oxide composition.

Oxide components of specimens were detected by X‐ray fluorescence (Rigaku Supermini200, Japan).

The mineral composition of samples was detected by X‐ray diffraction (Rigaku Smart‐Lab), under the conditions of Cu‐Kα radiation (λ = 1.54 Å), 45 kV, 200 mA, 2θ ranging from 5° to 70°, a scanning rate of 5°/min, and a step of 0.02°. To quantify the mineral content, the Rietveld analysis was conducted. 10 wt.% zinc oxide served as the internal standard and was introduced into the samples.

Thermogravimetric analysis (Rigaku Thermo plus EVO2) coupled with derivative thermogravimetry was conducted, where samples were heated from 50°C to 900°C at a rate of 10°C/min under a N_2_ atmosphere.

The structure of silicon compounds was determined by nuclear magnetic resonance (JEOL ECZ500R), with a spinning speed of 4 kHz, a spin relaxation of 1024 recycle times, a recycle delay of 30 s, and a scan of 500 times.

The morphology of the samples was detected by a scanning electron microscope (Tescan VEGA 3) at a voltage of 20 kV. An energy‐dispersive X‐ray spectrometer was equipped to conduct elemental analysis. Gold was coated on the sample surface to ensure conductivity.

Transmission electron microscopy (Talos F200S G2) was also used to observe the morphology with the Fast Fourier Transform analysis. The specimen was prepared by dilution in ethanol and placed on a carbon‐coated copper grid.

The particle size distribution of specimens was measured by Malvern Mastersizer 3000.

The compressive strength of specimens was evaluated by a servo‐hydraulic compression testing machine (WANCE HCT106A).

### 4.3 Electrochemical‐Driven Recycling Strategy

The electrochemical module was designed to produce acidic and alkaline solutions for subsequent concrete waste treatment. A custom‐built BPED system was constructed using a membrane stack composed of BPM, CEM, and AEM, forming the acid chamber, salt chamber, and base chamber, respectively. Each chamber was connected to a dedicated peristaltic pump to maintain continuous circulation of solutions and ensure homogeneous ion transport conditions. The platinum electrode (100 × 125 × 0.5 mm) was used due to its excellent resistance to acid and alkaline corrosion, as well as its high catalytic activity for redox reactions. The electrolyte was a 1 mol/L NaNO_3_ solution, and a constant voltage of 2 V was applied across the electrodes. The Na^+^ migration over time was monitored by ICP‐OES. The produced HNO_3_ and NaOH solutions were collected and stored for use in the subsequent waste treatment process.

The treatment module consists of a two‐step process designed to selectively extract and recover calcium‐ and silica‐based components from concrete waste, utilizing the acid and base solutions generated via the BPED module.

Step I—Dissolution of hydrated phases and precipitation of hydroxide: Each 20 g sample of concrete waste was added to a beaker. 1 mol/L HNO_3_ was introduced in varying volumes to achieve a Molar/Mass ratio from 1 to 5 mol/kg (increment: 1 mol/kg), followed by taking rotation of 150 rpm for 5 h. 0.2 mL suspension was extracted at selected time intervals analysis of metal ion concentration via ICP‐OES. The final pH was detected using a calibrated digital pH meter (PHBJ‐260) with standard buffers (pH 4.00, 6.86, and 9.18). The optimal Molar/Mass was determined by the recycling ratio‐time profiles of Ca. The suspension was vacuum‐filtered to obtain the solid residue and a metal‐rich filtrate. The residue was reserved for Step II. To recover metal ions from the filtrate, NaOH solution was added dropwise while monitoring the pH until it reached 12.5, resulting in the formation of a milky white suspension. The mixture was left undisturbed for 6 h to allow sedimentation (Figure S2A), then filtered. The precipitate was washed with DI water and isopropyl alcohol, then dried in a vacuum oven at 50°C and referred to as hydroxide precipitation.
Step II—Electrochemical extraction and precipitation of silica: The residue obtained from Step I was further processed for silica extraction. NaOH solutions with varying concentrations (0.25 to 1.25 mol/L, increment: 0.25 mol/L) were used at a fixed solid‐to‐liquid ratio of 1. The extraction was carried out at an elevated temperature of 90°C for 12 h with magnetic stirring. After filtration, the silicate‐rich solution was neutralized with HNO_3_ until the pH reached 7, initiating silica precipitation. The mixture was then rotated at 50 rpm for 24 h, followed by aging for 48 h. The resulting white powder, noted as silica precipitation (Figure S2B), was obtained through a series of processes of filtration, DI water washing, and drying at 105°C for 12 h. The optimal condition of silica extraction was determined by the silica yield‐molar concentration curve.

The remaining solid residue after silica extraction was collected and classified as electrochemical‐recycled granules (Figure S3), which were sieved into three size categories: powder (ER‐P, 0–0.3 mm), sand (ER‐S, 0.3–2.36 mm), and aggregate (ER‐A, 2.36–10 mm).

In the pilot‐scale study, real‐world concrete waste was treated under the optimized ER strategy developed at laboratory scale, using an integrated pilot system with an annual capacity of 19.2 tonnes. Molar/mass control and pH regulation were achieved using combined feed‐forward dosing and real‐time monitoring. The concentration of the acid was verified by inline conductivity measurement, while solution addition was controlled by a metering pump (0.01 L/min resolution) coupled with inline pH monitoring to maintain the target reaction window. NaNO_3_ functions as a recyclable electrolyte and is retained in the aqueous phase for reuse in subsequent BPED cycles. The process enabled the selective recovery of three product streams: hydroxide(HK), silica(HK), and electrochemical‐recycled granules(HK), classified into powder (ER‐P(HK), 0‐0.3 mm), sand (ER‐S(HK), 0.3–2.36 mm), and aggregate (ER‐A(HK), 2.36–20 mm). These recovered products were further characterized and used to fabricate RPC and RC, as described in subsequent sections.

### 4.4 Production of Reversed Concrete

Hydroxide and silica precipitation were exclusively utilized to synthesize reversed Portland clinker (r‐Pcl) for evaluating their viability as feedstock to completely replace natural mineral resources like limestone and clay in traditional cement production. The raw feed design, detailed in Appendix SB, was developed with a lime saturation factor (LSF) of 99.48, silica modulus (SM) of 2.04, and aluminium ratio (AR) of 0.84. According to the oxide composition and loss of ignition of the hydroxide and silica precipitations (Table S2), the mass ratio of hydroxide and silica precipitations for synthesizing r‐Pcl was 88.15:11.85. The two raw materials were mixed homogeneously using a V‐shape blender, and then placed in a crucible and heated at 1450°C with a rate of 5°C/min in a muffle furnace (Carbolite BLF1700) for 30 min. A rapid cooling method was conducted to avoid the decomposition of alite. After the bulk clinker was ground in an agate bowl, gypsum (5 wt.%) was added to the clinker to obtain reversed Portland Cement (RPC). Cement paste specimens using RPC and OPC were respectively fabricated under a fixed water/cement ratio of 0.4. Demoulding samples were conducted after 24 h of casting, followed by curing in saturated lime solution at 25°C till the age of 28 days (28d). The reutilizing feasibility of electrochemical‐recycled granules was verified by the strength of concrete, produced according to the mixture design in Table S8. The concrete specimen of 40 × 40 × 40 mm was fabricated using OPC and granules from different sources, including standard sand and natural aggregate (N‐C), demolished concrete waste without treatment (D‐C), and electrochemical‐recycled granules (ER‐C). After casting for 24 h, the samples were demoulded and subsequently cured in saturated lime solution at 25°C for 27 days.

In the pilot scale case study, the design parameters for reversed Portland clinker (r‐Pcl(HK)) synthesis were set as LSF: 98.31, SM: 1.57, and AR: 1.19 due to the varied oxide components of the feedstock. The same manufacturing process was conducted, and then finally obtained reversed Portland Cement (RPC(HK)). A cubic reversed concrete (RC(HK)) with the mixture proportion described in Table S8 was produced to prove the concept of fully utilizing recovered substances, including RPC(HK) and electrochemical‐recycled granules(HK). The same concrete size and curing methods were utilized for RC(HK).

### 4.5 Environmental and Economic Assessment

The environmental impacts of cement making and concrete systems, whether using traditional methods or ER technology, were assessed by the Life‐cycle analysis (LCA). The assessments were conducted in OpenLCA software, with the Ecoinvent database and relevant references as the inventory source. The system boundary was defined as cradle to gate for cement making and cradle to grave for concrete. Two functional units were selected: 1 t of cement and 1 m^3^ of concrete. Importantly, the inventory for the ER strategy was based on data from pilot‐scale implementation.

In parallel, an economic comparison was conducted between the conventional and ER‐based concrete routes, focusing on the net operating cost. This includes process‐related expenditures (e.g., electricity, fuel, raw materials, and carbon pricing). End‐of‐life waste management was handled differently: N‐C incurred landfill disposal costs based on regional tipping fees, whereas RC received economic credits for avoided landfilling. This focused approach avoids uncertainties related to capital expenditure and long‐term market fluctuations. Further details regarding the LCA and net operating cost calculations are depicted in Appendix SC.

## Author Contributions

Dr. Hanxiong Lyu conceptualized the paper, designed the experimental program, finished the experimental work, conducted the analysis, and wrote the draft manuscript. Mr. Zhenlin Li, Dr. Yang Liu, Dr. Lu Zhu, Dr. Lucen Hao, and Mr. Mingxin Shi contributed to the experimental work. Dr. Shipeng Zhang and Prof. Chi Sun Poon revised the draft manuscript and supervised the entire process.

## Funding

National Natural Science Foundation of China (Grant No. 52308283).

## Conflicts of Interest

The authors declare no conflicts of interest.

## Supporting information




**Supporting File**: advs74466‐sup‐0001‐SuppMat.docx.

## Data Availability

The data provided within this article and its Supplementary Information are available from the corresponding authors upon reasonable request.

## References

[1] P. Peduzzi , “Sand, Rarer Than One Thinks,” Environmental Development 11, no. 208–218 (2014): 682.

[2] M. Jaganmohan , Production Volume of Cement Worldwide From 1995 to 2023, (Statista, 2024), https://www.statista.com/statistics/1087115/global‐cement‐production‐volume.

[3] S. Frauke , K. Ioanna , S. B. Maria , R. Serge , and D. S. Luis , “Best Available Techniques (BAT) Reference Document for the Production of Cement, Lime and Magnesium Oxide, Industrial Emissions Directive 2010/75/EU (integrated pollution prevention and control),” in European Commission Joint Research Centre Institute for Prospective Technological Studies (Publications Office, 2013), https://doi/10.2788/12850.

[4] X. Li , H. Grassl , C. Hesse , and J. Dengler , “Unlocking the Potential of Ordinary Portland Cement With Hydration Control Additive Enabling Low‐Carbon Building Materials,” Communications Materials 5, no. 1 (2024): 1, 10.1038/s43246-023-00441-9.

[5] M. Pisciotta , H. Pilorgé , J. Davids , and P. Psarras , “Opportunities for Cement Decarbonization,” Cleaner Engineering and Technology 15 (2023): 100667, 10.1016/j.clet.2023.100667.

[6] E. Rigo , G. P. Gava , E. F. Felix , P. M. Borges , and E. Possan , “Concrete With Recycled Aggregates From Construction: Properties, Emissions and Carbon Capture assessment,” Case Studies in Construction Materials 23 (2025): e04983, 10.1016/j.cscm.2025.e04983.

[7] T. Wu , S. T. Ng , and J. Chen , “Incorporating Carbon Capture and Storage in Decarbonizing China's Cement Sector,” Renewable and Sustainable Energy Reviews 209 (2025): 115098, 10.1016/j.rser.2024.115098.

[8] K. Chen , J. Wang , B. Yu , H. Wu , and J. Zhang , “Critical Evaluation of Construction and Demolition Waste and Associated Environmental Impacts: A Scientometric Analysis,” Journal of Cleaner Production 287 (2021): 125071, 10.1016/j.jclepro.2020.125071.

[9] A. Habibi , A. M. Ramezanianpour , and M. Mahdikhani , “RSM‐based Optimized Mix Design of Recycled Aggregate Concrete Containing Supplementary Cementitious Materials Based on Waste Generation and Global Warming Potential,” Resources, Conservation and Recycling 167 (2021): 105420, 10.1016/j.resconrec.2021.105420.

[10] Y. Jiang , P. Shen , and C. S. Poon , “Improving the Bonding Capacity of Recycled Concrete Aggregate by Creating a Reactive Shell With Aqueous Carbonation,” Construction and Building Materials 315 (2022): 125733, 10.1016/j.conbuildmat.2021.125733.

[11] N. Li and C. Unluer , “Enhancement of the Wet Carbonation of Artificial Recycled Concrete Aggregates in Seawater,” Cement and Concrete Research 175 (2024): 107387, 10.1016/j.cemconres.2023.107387.

[12] S. Jamil , J. Shi , and M. Idrees , “Effect of Various Parameters on Carbonation Treatment of Recycled Concrete Aggregate Using the Design of Experiment Method,” Construction and Building Materials 382 (2023): 131339, 10.1016/j.conbuildmat.2023.131339.

[13] S. K. Kaliyavaradhan , T.‐C. Ling , and K. H. Mo , “CO_2_ Sequestration of Fresh Concrete Slurry Waste: Optimization of CO_2_ Uptake and Feasible use as a Potential Cement Binder,” Journal of CO2 Utilization 42 (2020): 101330, 10.1016/j.jcou.2020.101330.

[14] N. K. Bui , T. Satomi , and H. Takahashi , “Mechanical Properties of Concrete Containing 100% Treated Coarse Recycled Concrete Aggregate,” Construction and Building Materials 163 (2018): 496–507, 10.1016/j.conbuildmat.2017.12.131.

[15] W.‐Z. Chen , C.‐J. Jiao , X.‐C. Zhang , Y. Yang , and X.‐F. Chen , “Study on the Microstructure of Recycled Aggregate Concrete Strengthened by the Nano‐SiO_2_ Soaking Method,” Structures 58 (2023): 105388, 10.1016/j.istruc.2023.105388.

[16] A. H. Siletani , S. Asayesh , A. A. Shirzadi Javid , A. Habibnejad Korayem , and M. A. Ghanbari , “Influence of Coating Recycled Aggregate Surface With Different Pozzolanic Slurries on Mechanical Performance, Durability, and Micro‐Structure Properties of Recycled Aggregate Concrete,” Journal of Building Engineering 83 (2024): 108457, 10.1016/j.jobe.2024.108457.

[17] W. F. Santos , M. Quattrone , V. M. John , and S. C. Angulo , “Roughness, Wettability and Water Absorption of Water Repellent Treated Recycled Aggregates,” Construction and Building Materials 146 (2017): 502–513, 10.1016/j.conbuildmat.2017.04.012.

[18] G. Sua‐iam and N. Makul , “Self‐Compacting Concrete Produced With Recycled Concrete Aggregate Coated by a Polymer‐Based Agent: A Case Study,” Case Studies in Construction Materials 19 (2023): e02351, 10.1016/j.cscm.2023.e02351.

[19] J. Nilimaa , “Smart Materials and Technologies for Sustainable Concrete Construction,” Developments in the Built Environment 15 (2023): 100177, 10.1016/j.dibe.2023.100177.

[20] A. Gadayev and B. Kodess , “By‐Product Materials in Cement Clinker Manufacturing,” Cement and Concrete Research 29, no. 2 (1999): 187–191, 10.1016/S0008-8846(98)00094-5.

[21] Y. R. Patil , V. A. Dakwale , and R. V. Ralegaonkar , “Recycling Construction and Demolition Waste in the Sector of Construction,” Advances in Civil Engineering 2024, no. 1 (2024): 6234010, 10.1155/2024/6234010.

[22] T. Watari , Z. Cao , S. Hata , and K. Nansai , “Efficient Use of Cement and Concrete to Reduce Reliance on Supply‐Side Technologies for Net‐Zero Emissions,” Nature Communications 13, no. 1 (2022): 4158, 10.1038/s41467-022-31806-2. PMC9293885 35851585

[23] L. D. Ellis , A. F. Badel , M. L. Chiang , R. J.‐Y. Park , and Y.‐M. Chiang , “Toward Electrochemical Synthesis of Cement—An Electrolyzer‐Based Process for Decarbonating CaCO_3_ While Producing Useful Gas Streams,” Proceedings of the National Academy of Sciences 117, no. 23 (2020): 12584–12591, 10.1073/pnas.1821673116. PMC7293631 31527245

[24] N. Bahnasawy , S. Al Anany , and N. K. Allam , “Electrochemical Catalysis for the Production of Green Cement: Towards Decarbonizing the Cement Industry,” Catalysis Science & Technology 14, no. 15 (2024): 4087–4105, 10.1039/D4CY00009A.

[25] Z. Zhang , A. S. R. Williams , S. Ren , et al., “Electrolytic Cement Clinker Precursor Production Sustained Through Orthogonalization of Ion Vectors,” Energy & Environmental Science 18, no. 5 (2025): 2395–2404, 10.1039/D4EE04881D.

[26] X. K. Lu , W. Zhang , B. N. Ruggiero , L. C. Seitz , and J. Li , “Scalable Electrified Cementitious Materials Production and Recycling,” Energy & Environmental Science 17, no. 24 (2024): 9566–9579, 10.1039/D4EE03529A.

[27] T. Ji , S. Ren , and Y. Yang , “Low‐Emission Cement Clinker Precursor Production, Enabled by Electrolytic Extraction of Calcium From Waste Cement,” Nature Communications 16, no. 1 (2025): 9302, 10.1038/s41467-025-64339-5. PMC12541047 41120382

[28] K. B. Krauskopf , “Dissolution and Precipitation of Silica at Low Temperatures,” Geochimica et Cosmochimica Acta 10, no. 1 (1956): 1–26, 10.1016/0016-7037(56)90009-6.

[29] F. K. Crundwell , “The Mechanism of Dissolution of Minerals in Acidic and Alkaline Solutions: Part II Application of a New Theory to Silicates, Aluminosilicates and Quartz,” Hydrometallurgy 149 (2014): 265–275, 10.1016/j.hydromet.2014.07.003.

[30] T. H. Elmer and M. E. Nordberg , “Solubility of Silica in Nitric Acid Solutions,” Journal of the American Ceramic Society 41, no. 12 (1958): 517–520, 10.1111/j.1151-2916.1958.tb12907.x.

[31] F. Wang , G. Long , M. Bai , et al., “A New Perspective on Belite‐ye'elimite‐ferrite Cement Manufactured From Electrolytic Manganese Residue: Production, Properties, and Environmental Analysis,” Cement and Concrete Research 163 (2023): 107019, 10.1016/j.cemconres.2022.107019.

[32] N. Koga , S. Takemoto , S. Okada , and H. Tanaka , “A Kinetic Study of the Thermal Decomposition of Iron(III) Hydroxide Oxides. Part 1. α‐FeO(OH) in Banded Iron Formations,” Thermochimica Acta 254 (1995): 193–207, 10.1016/0040-6031(94)02050-X.

[33] V. K. Singh , “12 – Hydration and setting of Portland cement,” in The Science and Technology of Cement and Other Hydraulic Binders, ed. V. K. Singh , (Woodhead Publishing, 2023), 423–466.

[34] D. Wang , Q. Wang , and Z. Huang , “Reuse of Copper Slag as a Supplementary Cementitious Material: Reactivity and Safety,” Resources, Conservation and Recycling 162 (2020): 105037, 10.1016/j.resconrec.2020.105037.

[35] E. B. Nelson , in Well Cementing (Newnes, 1990).

[36] F. Vázquez‐Acosta , L. M. Torres‐Martínez , W. López‐González , and J. Ibarra‐Rodríguez , “Influence of Iron Content on the Color of the C_3_A–Fe_2_O_3_ System Synthesized Under Different Conditions of Temperature, Atmosphere and Cooling,” Ceramics International 38, no. 4 (2012): 3261–3272, 10.1016/j.ceramint.2011.12.032.

[37] A. Baral , C. Pesce , A. S. Yorkshire , et al., “Characterisation of Iron‐Rich Cementitious Materials,” Cement and Concrete Research 177 (2024): 107419, 10.1016/j.cemconres.2023.107419.

[38] G. Prokopski and J. Halbiniak , “Interfacial Transition Zone in Cementitious Materials,” Cement and Concrete Research 30, no. 4 (2000): 579–583, 10.1016/S0008-8846(00)00210-6.

[39] A. Chajec , “The Use of Granite Powder Waste in Cementitious Composites,” Journal of Materials Research and Technology 25 (2023): 4761–4783, 10.1016/j.jmrt.2023.06.253.

[40] P. Jagadesh , S. Oyebisi , A. H. Muthu , A. Sarulatha , K. Supikshaa , and V. P. Vhishva laxmy , “Microscopic Analyses and Performance Characteristics of Granite Powder Blended Cement,” Construction and Building Materials 426 (2024): 136006, 10.1016/j.conbuildmat.2024.136006.

[41] A. Zhou , G. Miao , Y. Zhang , et al., “Effects of Stone Powder on Microstructure of Manufactured‐sand Concrete and Its Gas Permeability,” Journal of Building Engineering 91 (2024): 109539, 10.1016/j.jobe.2024.109539.

[42] H. Mikulčić , M. Vujanović , D. K. Fidaros , et al., “The Application of CFD Modelling to Support the Reduction of CO_2_ Emissions in Cement Industry,” Energy 45, no. 1 (2012): 464–473, 10.1016/j.energy.2012.04.030.

[43] C. F. Dunant , S. Joseph , R. Prajapati , and J. M. Allwood , “Electric Recycling of Portland Cement at Scale,” Nature 629 (2024): 1055–1061, 10.1038/s41586-024-07338-8. 38778099 PMC11136652

[44] J. Jaskowska‐Lemanska , “Impurities of Recycled Concrete Aggregate‐types, Origin and Influence on the Concrete Strength Parameters,” in IOP Conference Series: Materials Science and Engineering (IOP Publishing, 2019), 042056.

[45] Z. H. Duan and C. S. Poon , “Properties of Recycled Aggregate Concrete Made With Recycled Aggregates With Different Amounts of Old Adhered Mortars,” Materials & Design 58 (2014): 19–29, 10.1016/j.matdes.2014.01.044.

[46] H. CoP, Code of Practice for Structural Use of Concrete 2013, in Buildings Department (BD) (Buildings Department, 2013).

[47] Energy & emissions projections 2050 – EnerOutlook. Enerdata, 2024, https://eneroutlook.enerdata.net/forecast‐world‐co2‐intensity‐of‐electricity‐generation.html.

[48] Z. Zong , O. Daoud , N. P. Hankins , Q. She , and C. D. Peters , “Valorising Desalination Brine for Green Cement Production: Toward Mitigating Global CO_2_ Emissions,” Water Research 284 (2025): 123930, 10.1016/j.watres.2025.123930. 40494198

[49] R. Feiz , J. Ammenberg , L. Baas , M. Eklund , A. Helgstrand , and R. Marshall , “Improving the CO_2_ Performance of Cement, Part I: Utilizing Life‐Cycle Assessment and Key Performance Indicators to Assess Development Within the Cement Industry,” Journal of Cleaner Production 98 (2015): 272–281, 10.1016/j.jclepro.2014.01.083.

[50] M. B. Ali , R. Saidur , and M. S. Hossain , “A Review on Emission Analysis in Cement Industries,” Renewable and Sustainable Energy Reviews 15, no. 5 (2011): 2252–2261, 10.1016/j.rser.2011.02.014.

[51] K. Scrivener , F. Martirena , S. Bishnoi , and S. Maity , “Calcined Clay Limestone Cements (LC^3^),” Cement and Concrete Research 114 (2018): 49–56, 10.1016/j.cemconres.2017.08.017.

[52] M. A. Jungclaus , S. L. Williams , J. H. Arehart , and W. V. Srubar , “Whole‐life Carbon Emissions of Concrete Mixtures Considering Maximum CO_2_ Sequestration via Carbonation,” Resources, Conservation and Recycling 206 (2024): 107605, 10.1016/j.resconrec.2024.107605.

[53] F. Xi , S. J. Davis , P. Ciais , et al., “Substantial Global Carbon Uptake by Cement Carbonation,” Nature Geoscience 9, no. 12 (2016): 880–883, 10.1038/ngeo2840.

[54] Z. Cao , R. J. Myers , R. C. Lupton , et al., “The Sponge Effect and Carbon Emission Mitigation Potentials of the Global Cement Cycle,” Nature Communications 11, no. 1 (2020): 3777, 10.1038/s41467-020-17583-w. PMC7392754 32728073

