# Supplementary material for: Scalable Electrochemical Concrete Fabrication and Closed‐Loop Recycling
Source: Adv Sci (Weinh). 2026 Feb 25;13(24):e23927. doi: 10.1002/advs.202523927 (PMC13116295; doi:10.1002/advs.202523927)
Supplement: Supplementary file 1 — Supporting File: advs74466‐sup‐0001‐SuppMat.docx. [file ADVS-13-e23927-s001.docx]

Supporting Information

**Scalable Electrochemical Concrete Fabrication and Closed-loop Recycling**

*Hanxiong Lyu,* *Zhenlin Li, Yang Liu, Lu Zhu, Lucen Hao, Mingxin Shi, Shipeng Zhang*, Chi Sun Poon*

* Corresponding author: Shipeng Zhang

Email: shipeng.zhang@polyu.edu.hk

**This file includes:**

Figure S1 to S9

Tables S1 to S20

Supplementary Appendix A to D

SI References


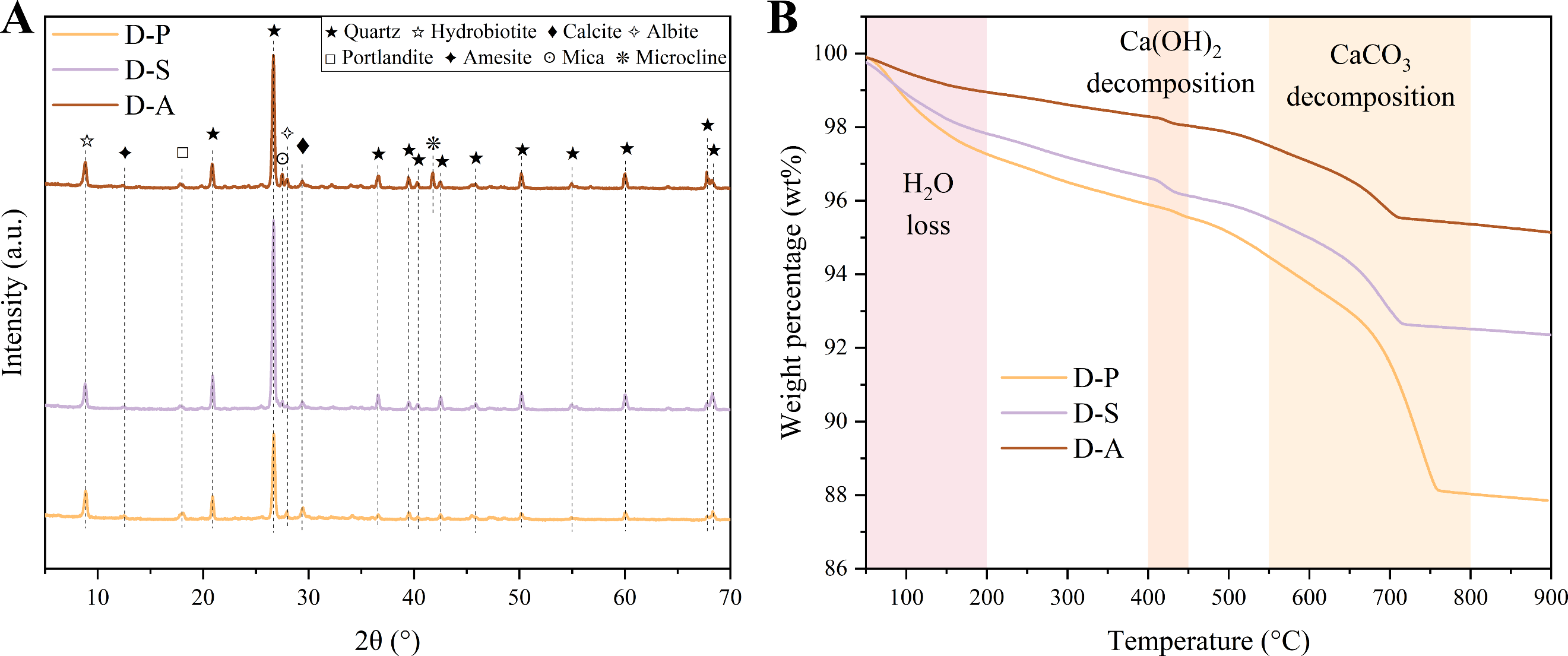


**Figure S1** **Characterizations of demolished concrete waste, including powder (D-P), sand (D-S), and aggregate (D-A).** (**A**) X-ray diffraction analysis. (**B**) Thermogravimetric analysis. (Several mineral components are identified in D-P, D-S, and D-A, including quartz, hydrobiotite, albite, amesite, mica, and microcline, inherited from the standard sand and natural aggregates used in the parent concrete. Additionally, the cement paste residuals adhering to the sand and aggregate particles are composed primarily of amorphous calcium silicate hydrates, portlandite, and calcite, confirmed by both X-ray diffraction analysis and thermogravimetric analysis. The thermal decomposition temperatures of these compounds are, respectively, as follows: 50-200 °C for calcium silicate hydrate, 400-450 °C for portlandite, and 550-800 °C for calcite ^1, 2^.)

**Table S1.** Oxide components of D-P, D-S, and D-A [wt%].

|  | D-P | D-S | D-A |
| --- | --- | --- | --- |
| CaO | 36.9 | 22.1 | 19.8 |
| SiO_2_ | 41.5 | 58.6 | 58.2 |
| Al_2_O_3_ | 7.97 | 7.43 | 8.79 |
| Fe_2_O_3_ | 5.79 | 4.59 | 5.25 |
| Na_2_O | 0.661 | 0.629 | 0.876 |
| MgO | 2.02 | 1.73 | 1.76 |
| K_2_O | 2.79 | 2.91 | 3.3 |
| SO_3_ | 1.34 | 0.952 | 0.802 |
| TiO_2_ | 0.602 | 0.63 | 0.875 |
| Others | 0.427 | 0.429 | 0.347 |


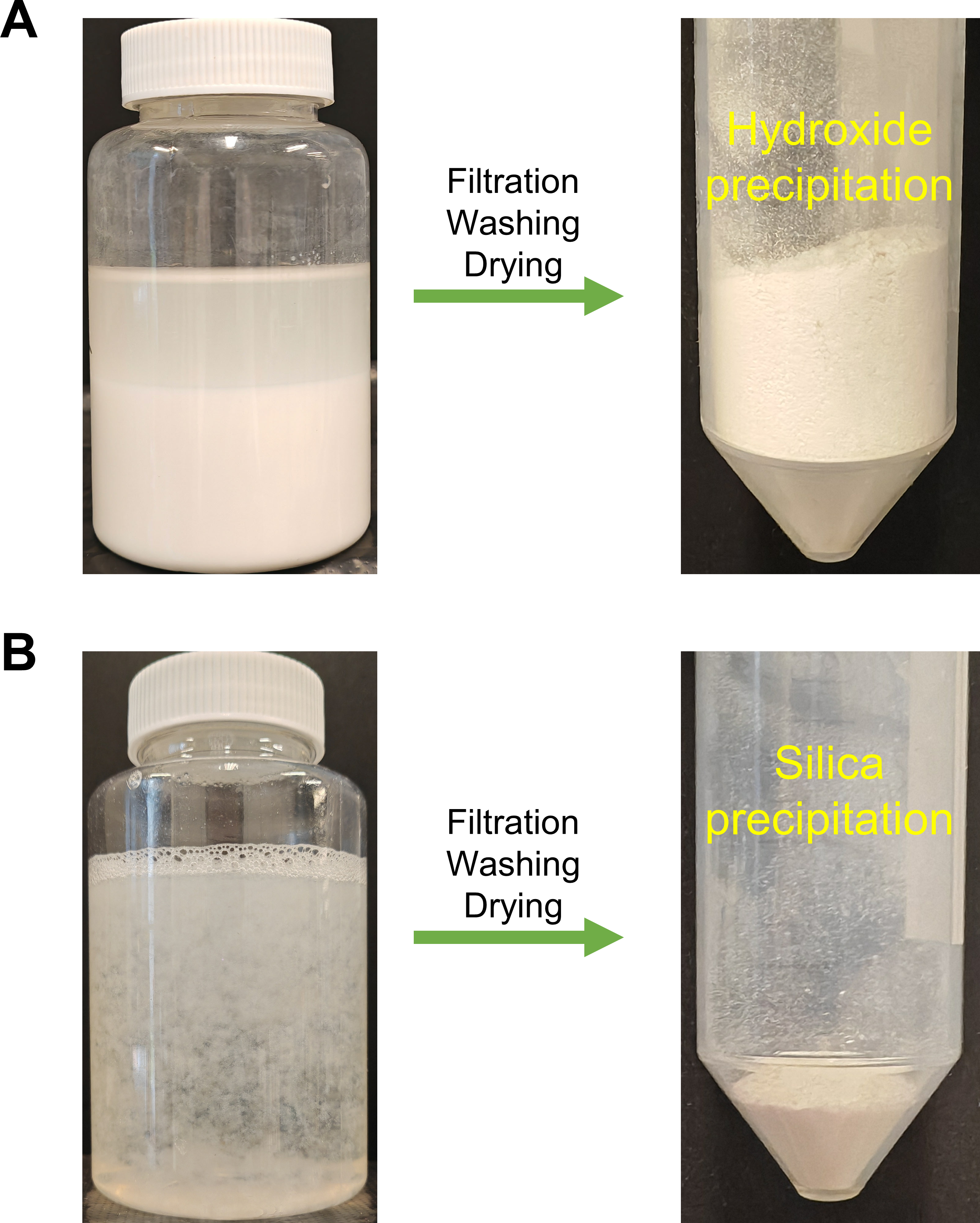


**Figure S2 Visual image of hydroxide and silica precipitation.** (A) Step I and (B) Step II.


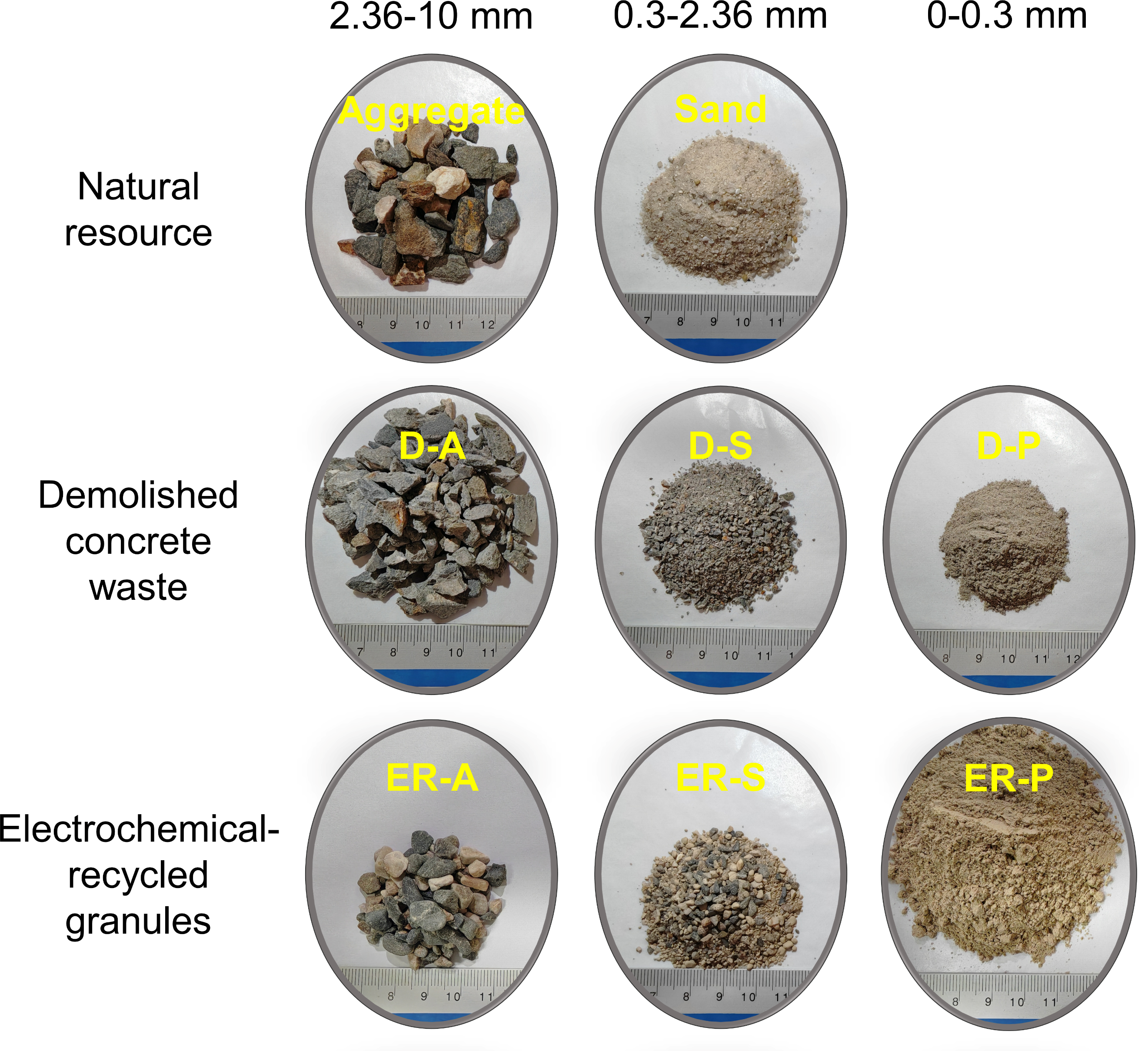


**Figure S3** **Visual image of aggregate from natural resources, demolished concrete waste, and electrochemical-recycled granules with different size ranges.** (Demolished concrete waste includes powder (D-P), sand (D-S), and aggregate (D-A), and electrochemical-recycled granules containing powder (ER-P), sand (ER-S), and aggregate (ER-A).)


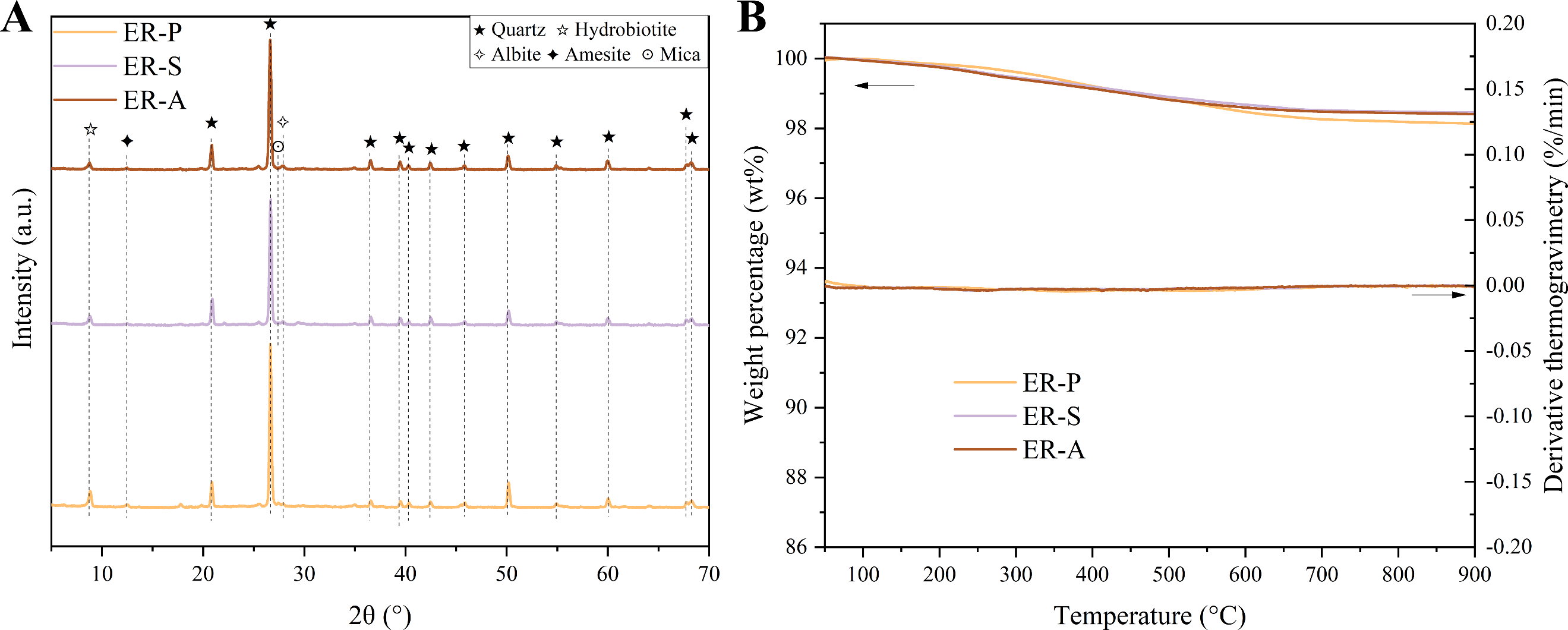


**Figure S4** **Characterizations of electrochemical-recycled granules, including powder (ER-P), sand (ER-S), and aggregate (ER-A).** (**A**) X-ray diffraction patterns; (**B**) Thermogravimetric and differential thermogravimetric analysis of ER-P, ER-S, and ER-A. (Besides the mineral phases inherited from the spent aggregates in the parent concrete, no portlandite and calcite are detected, also verified by the thermogravimetric analysis and derivative thermogravimetry results.)

**CO_2_ mineralization technology**

Due to the weathering carbonation over the service life, the concrete waste contains a small quantity of calcium carbonate.

In Step I, the acidic environment causes the calcium carbonate to decompose, releasing Ca^2+^ and CO_2_ gas. Advanced CO_2_ mineralization technology offers a promising solution by injecting CO_2_ gas into the calcium-rich solutions ^3^. The formation of calcite indicates that CO_2_ can be mineralized through the reaction with Ca^2+^.


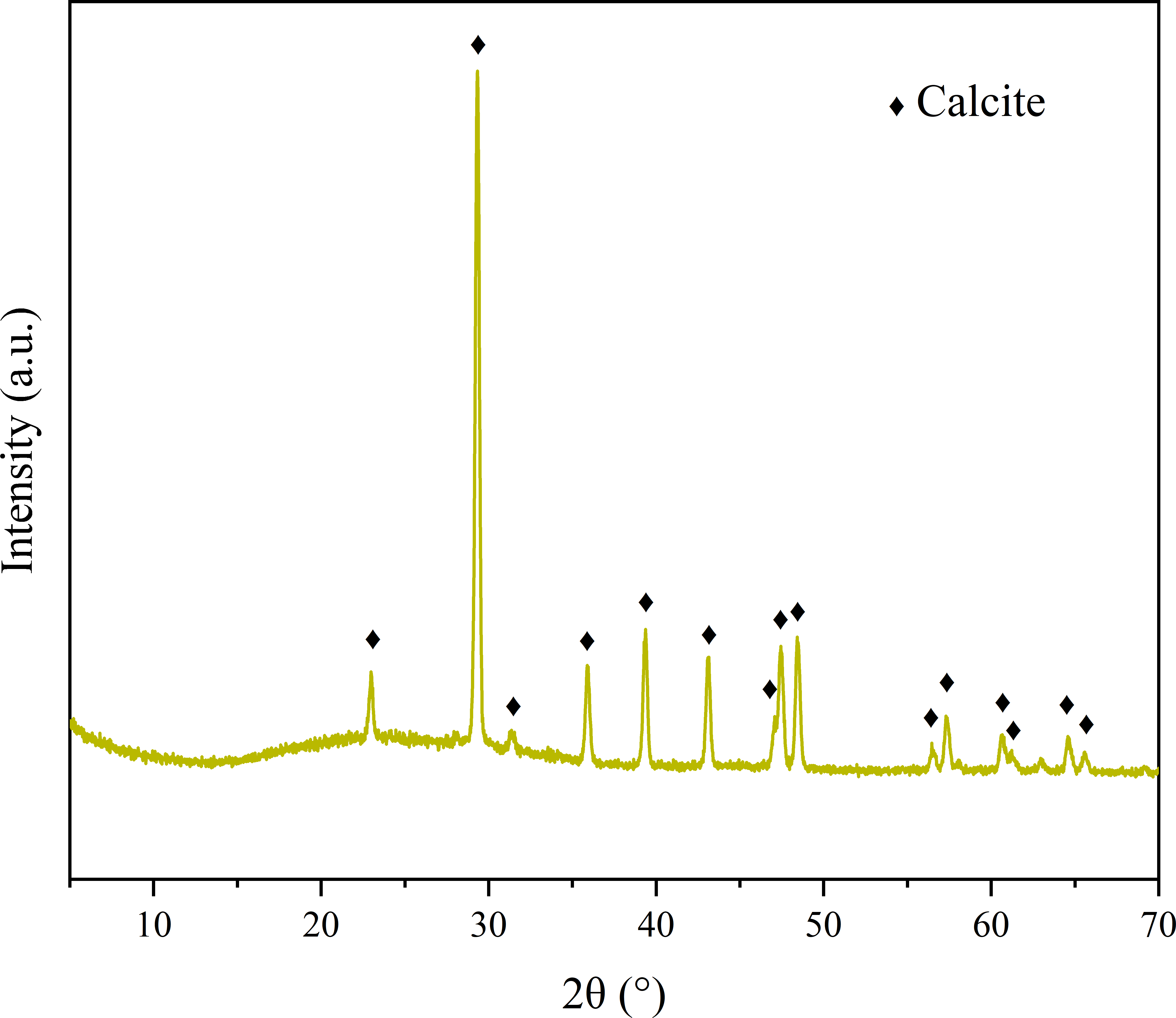


**Figure S5** **X-ray diffraction pattern of the product fabricated by injecting the CO_2_ gas into calcium-rich solutions.**

**Table S2.** Oxide components and loss of ignition (LOI) of hydroxide precipitation and silica precipitation [wt%].

|  | | Hydroxide precipitation | Silica precipitation |
| --- | --- | --- | --- |
| CaO | | 76.9 | 1.15 |
| SiO_2_ | | 6.69 | 96.3 |
| Al_2_O_3_ | | 5.09 | 1.43 |
| Fe_2_O_3_ | | 6.32 | 0.26 |
| MgO | | 3.27 | 0 |
| Others | K_2_O | 0.0587 | 0.2 |
|  | SO_3_ | 0.801 | 0.63 |
|  | TiO_2_ | 0.35 | 0 |
|  | P_2_O_5_ | 0.238 | 0 |
|  | MnO | 0.103 | 0 |
|  | NiO | 0.026 | 0 |
|  | CuO | 0.0467 | 0 |
|  | ZnO | 0.0728 | 0.0285 |
|  | SrO | 0.0468 | 0 |
| LOI (950 °C) ^a)^ | | 26.63% | 2.13% |

(Note: ^a)^ LOI (950 °C) corresponds to the loss of ignition at 950 °C for 1 h.)

**Table S3.** Particle size distribution and specific surface area of hydroxide and silica precipitation.

|  | D10 [μm] | D50 [μm] | D90 [μm] | Specific surface area [m^2^/kg] |
| --- | --- | --- | --- | --- |
| Hydroxide precipitation | 7.09 | 37.67 | 112.18 | 341.9 |
| Silica precipitation | 7.83 | 35.50 | 117.24 | 304.5 |

**Table S4** Chemical oxide composition of RPC and OPC [wt%]

|  | RPC | OPC |
| --- | --- | --- |
| CaO | 64.2 | 68.5 |
| SiO_2_ | 19.3 | 15.5 |
| Al_2_O_3_ | 4.3 | 4.31 |
| Fe_2_O_3_ | 5.13 | 4.45 |
| MgO | 2.63 | 1.58 |
| K_2_O | 0.08 | 0.656 |
| SO_3_ | 3.68 | 4.18 |
| TiO_2_ | 0.282 | 0.399 |
| Others | 0.398 | 0.425 |

**Table S5.** Rietveld refinement results of X-ray diffraction patterns for RPC and OPC [%].

|  | Alite | Belite | Tricalcium aluminate | Brownmillerite | Gypsum | Lime | Amorphous | Goodness of Fit |
| --- | --- | --- | --- | --- | --- | --- | --- | --- |
| RPC | 48.8 | 32.7 | 3.1 | 4.7 | 5.0 | 0.7 | 4.9 | 1.73 |
| OPC | 49.4 | 30.0 | 4.4 | 4.7 | 5.0 | - | 6.6 | 1.78 |


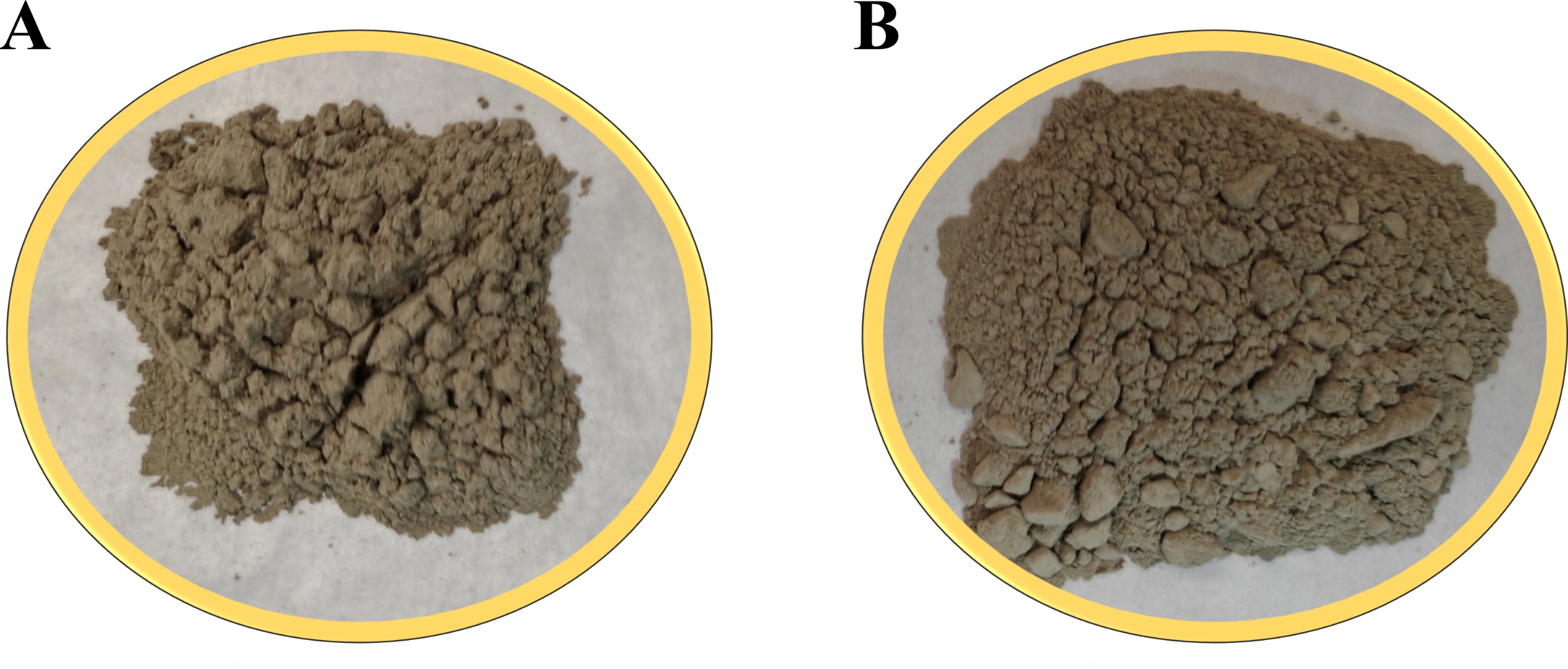


**Figure S6** **Optical image of (A)** RPC **and (B)** OPC**.**


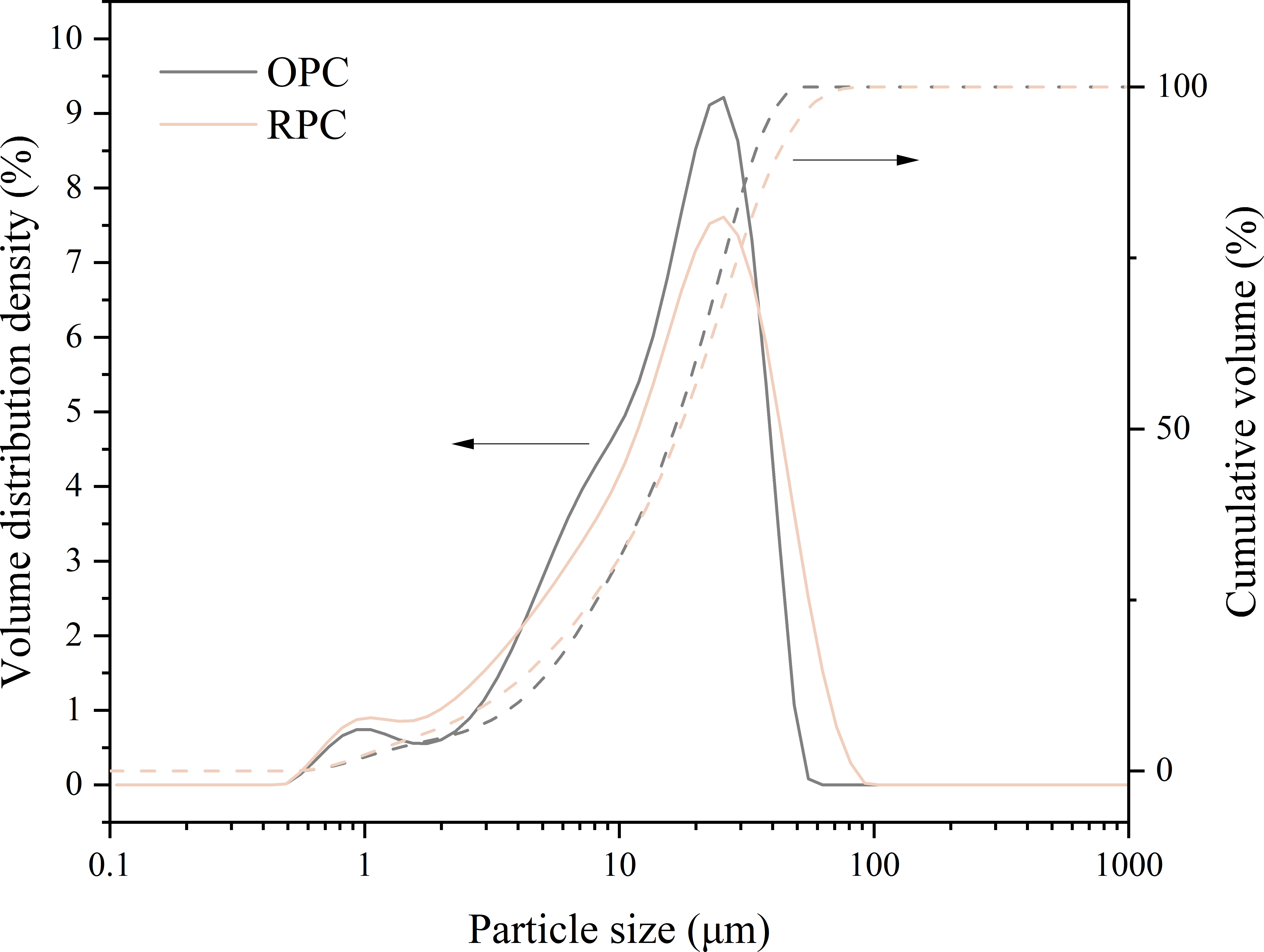


**Figure S7** **Particle size distribution of RPC and OPC.**

**Table S6.** Particle size distribution and specific surface area of RPC and OPC.

|  | D10 [μm] | D50 [μm] | D90 [μm] | Specific surface area [m^2^/kg] |
| --- | --- | --- | --- | --- |
| OPC | 4.01 | 16.49 | 33.85 | 784.9 |
| RPC | 3.07 | 17.28 | 41.77 | 854.8 |

**Table S7.** Water absorption ratio of sand and aggregates from different sources.

|  | Natural sources | Demolished concrete waste | Electrochemical- recycled granules |
| --- | --- | --- | --- |
| 0.3-2.36 mm (sand) | 2.3% | 11.76% | 3.53% |
| 2.36-10 mm (aggregate) | 0.7% | 8.89% | 1.09% |

**Table S8.** Mixture proportions of concrete [kg/m^3^].

|  | OPC | RPC(HK) | Fine  aggregate:  0.3-2.36 mm | Coarse aggregate:  2.36-10 mm | Coarse aggregate:  2.36-20 mm | Water |
| --- | --- | --- | --- | --- | --- | --- |
| N-C | 410 | - | 636  (Using standard sand) | 1131  (Using natural aggregate) | - | 164 |
| D-C |  |  | 636  (Using D-S) | 1131  (Using D-A) |  |  |
| ER-C |  |  | 636  (Using ER-S) | 1131  (Using ER-A) |  |  |
| RC(HK) | - | 410 | 636  (Using  ER-S(HK)) | - | 1131  (Using  ER-A(HK)) |  |

**Table S9.** Oxide components of electrochemical-recycled granules [wt%].

|  | ER-P | ER-S | ER-A |
| --- | --- | --- | --- |
| CaO | 1.67 | 1.57 | 0.69 |
| SiO_2_ | 70.78 | 64.7 | 68.0 |
| Al_2_O_3_ | 11.65 | 14.1 | 14.0 |
| Fe_2_O_3_ | 3.87 | 6.14 | 8.02 |
| Na_2_O | 4.39 | 3.05 | 0 |
| MgO | 0.44 | 2.75 | 2.65 |
| K_2_O | 6.01 | 6.23 | 4.98 |
| SO_3_ | 0.09 | 0.02 | 0.05 |
| TiO_2_ | 0.87 | 0.80 | 1.08 |
| Others | 0.23 | 0.64 | 0.53 |

**Table S10.** Circularity index of the ER strategy for 1 kg laboratory-simulated concrete wastes.

| Weight of input material [g] | |  | Weight of reversed substances [g] | | Circularity index (wt%) |
| --- | --- | --- | --- | --- | --- |
| Concrete waste | Concrete waste  heated to 900 °C |  | r-Pcl | Electrochemical-  recycled granules |  |
| 1000 | 935.8 |  | 103 | 781 | 94.5 |


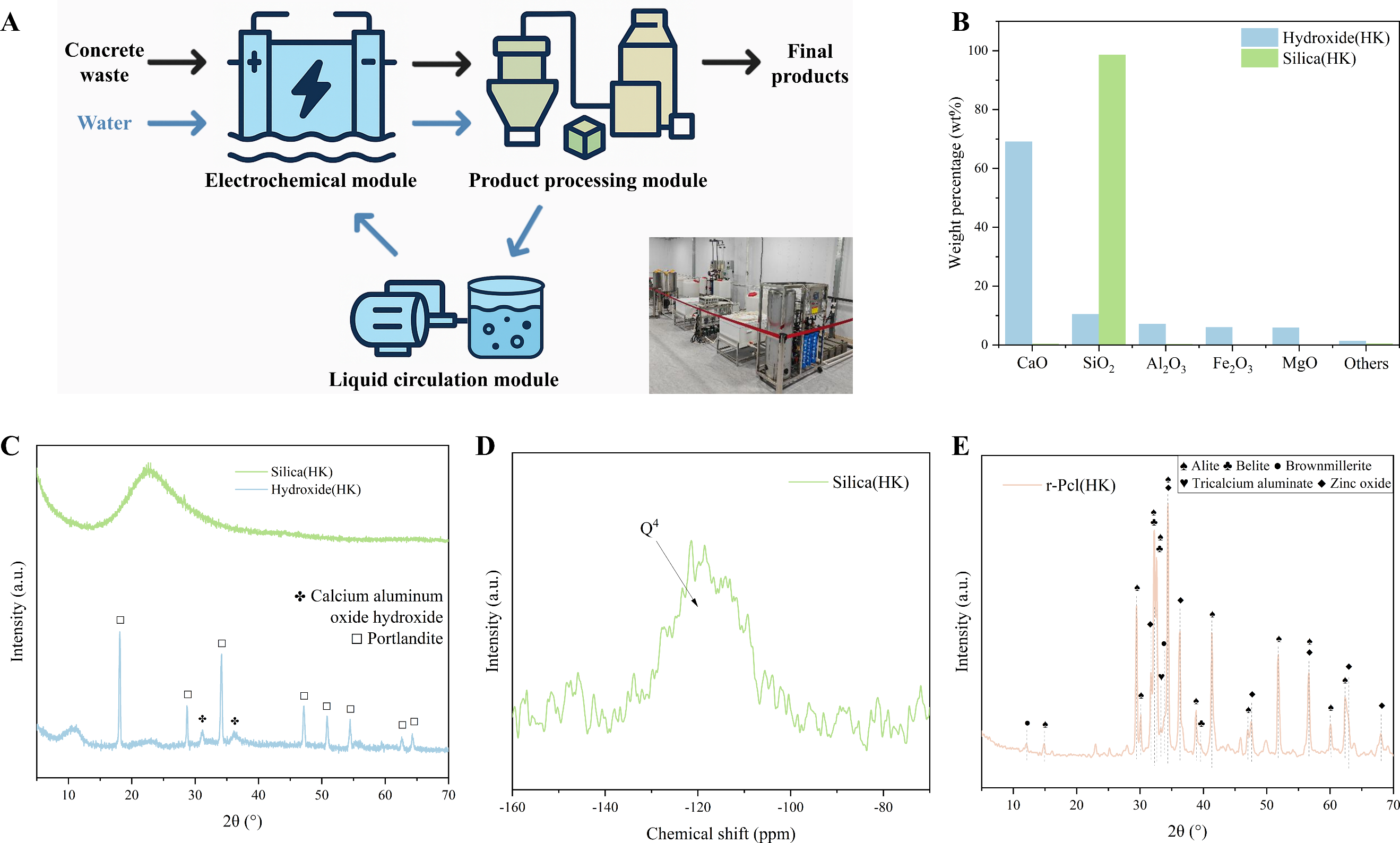


**Figure S8** **Pilot scale validation.** (**A**) ER system diagram and site photo. (**B**) Oxide components of hydroxide(HK) and silica(HK) examined by X-ray fluorescence. (**C**) X-ray diffraction patterns of hydroxide(HK) and silica(HK) with a goodness of fit of 0.79. (**D**) ^29^Si Nuclear magnetic resonance result of silica(HK). (**E**) X-ray diffraction pattern of r-Pcl(HK).

**Table S11.** Oxide components and loss of ignition (LOI) of hydroxide(HK) and silica(HK) [wt%].

|  | | Hydroxide(HK) | Silica(HK) |
| --- | --- | --- | --- |
| CaO | | 69.116 | 0.359 |
| SiO_2_ | | 10.48 | 98.6 |
| Al_2_O_3_ | | 7.137 | 0.314 |
| Fe_2_O_3_ | | 6.017 | 0.153 |
| MgO | | 5.872 | 0.106 |
| Others | K_2_O | 0 | 0.317 |
|  | SO_3_ | 0.302 | 0.0923 |
|  | Na_2_O | 0.281 | 0 |
|  | TiO_2_ | 0.227 | 0 |
|  | P_2_O_5_ | 0.252 | 0 |
|  | MnO | 0.139 | 0 |
|  | CuO | 0.03 | 0 |
|  | ZnO | 0.07 | 0 |
|  | SrO | 0.025 | 0 |
|  | Cl | 0.019 | 0.0426 |
|  | Rb_2_O | 0 | 0.0286 |
|  | PbO | 0.019 | 0 |
|  | NiO | 0.015 | 0 |
| LOI (500 °C) ^a)^ | | 25.19% | - |
| LOI (950 °C) ^b)^ | | 32.58% | 3.56% |

(Note: ^a)^ LOI (500 °C) corresponds to the loss of ignition at 500 °C for 1 h, to detect the mass of water loss caused by hydroxide decomposition; ^b)^ LOI (950 °C) corresponds to the loss of ignition at 950 °C for 1 h, to detect the total mass loss.)

**Table S12.** Weight ratio of each recycled substance to the total real-world concrete wastes [wt%].

| Hydroxide(HK) | Silica(HK) | ER-P(HK) | ER-S(HK) | ER-A(HK) | Others |
| --- | --- | --- | --- | --- | --- |
| 17.78 | 1.53 | 11.15 | 24.13 | 42.01 | 3.4 |

(Notes: The above value is calculated as the ratio of the mass of recycled substance to the total input material mass. The “Others” category represents the portion that cannot be recycled, such as bonded water and mineralized CO_2_ embodied in the concrete waste.)

**Table S13.** Circularity index of the ER strategy for 1 kg of real-world concrete waste.

| Weight of input material [g] | |  | Weight of reversed substances [g] | | Circularity index (wt%) |
| --- | --- | --- | --- | --- | --- |
| Concrete waste | Concrete waste  heated to 900 °C |  | r-Pcl(HK) | Electrochemical-  recycled granules(HK) |  |
| 1000 | 949.75 |  | 133.15 | 772.91 | 95.4 |

**Table S14.** Physical properties of D-P, D-S, and D-A.

|  | D-P | D-S | D-A |
| --- | --- | --- | --- |
| Density [g/cm^3^] ^a)^ | 2.5144 | 2.6254 | 2.64164 |
| Moisture content [wt%] ^b)^ | 3.80 | 2.20 | 1.56 |
| Residual cement paste [wt%] ^c)^ | 44.40 | 21.75 | 16.70 |

(Note: ^a)^ The density of demolished concrete wastes was examined by a gas intrusion pycnometer. ^b)^: The moisture content was determined by the mass difference before and after drying. ^c)^: The cement paste residues were determined by the acid dissolution method.)

**Supplementary Appendix A**: Current state of recycling concrete waste by advanced technologies

Concrete waste recycling has become increasingly critical in the past decade, driven by the need for sustainable construction practices and the rising awareness of reducing landfilling. As the world’s most widely used building material, concrete generates vast amounts of waste at the end of its service life. Numerous efforts have been made to tackle this challenge, and Appendix A outlines the key advancements in recycling concrete waste, highlighting the existing recycling strategies from 50 published articles over the last 10 years. These technologies include carbonation, chemical modification, acid soaking, thermal processing, pozzolanic slurry immersion, mechanical treatment, polymer impregnation, bio-deposition, microwave activation, sonication, and water washing. Among these, carbonation has emerged as one of the most prominent techniques in the past five years, listed in Table S15, representing 30% of the summarized studies. The remaining advanced technologies are summarized in Table S16. Despite great variations in terms of the source and composition of raw concrete wastes and experimental parameters between publications, some valuable patterns and insights can still be drawn.

Carbonation is a technique that utilizes greenhouse gas CO_2_ to convert the calcium-bearing phases (cement paste residues) in concrete waste into calcium carbonate (Cc), while permanent carbon sequestration can be achieved. This technology exhibits promising results with D-P, which is a marginal fraction of concrete waste with higher calcium content. However, for D-S and D-A, which cumulatively account for 50-90% of the processed concrete waste ^4^, the lower paste content in these fractions greatly reduces the efficiency of carbonation. Meanwhile, despite the primary products from carbonation (Cc and silica gel) having the potential to serve as reactive supplementary cementitious materials (SCMs) to partially replace OPC, their incorporation in new concrete mixes is relatively low, typically less than 40%. Besides, Cc and silica gel normally coexist in the carbonation product, necessitating an additional separation process if high purity of Cc or silica gel is pursued, which potentially leads to more chemical consumption and environmental concerns associated with additional chemical wastes.

In recycling technologies beyond carbonation, the primary objective is to remove or enhance the residual old paste on the surface of D-S and D-A, resulting in an artificial aggregate material suitable for producing new concrete. The old paste poses several issues: beyond its high-water absorption rate, it introduces micro-cracks and induces a multi-layered interfacial transition zone, which detrimentally affects the concrete. By conducting recycling technologies, the treated aggregates are primarily used as an alternative to natural aggregates, but their performance varies significantly, influenced by factors such as the effectiveness of the recycling process and the experimental conditions. Although aggregate can be successfully recovered, the existing practice fails to recover the cement from the paste residuals. It is undeniable that cement is the most carbon-intensive ingredient in concrete. Meanwhile, cement is also the most valuable part of concrete, completely overlooked by these practices. Only the downcycling process might not make economic sense.

Over the past decade, myriad endeavours have been undertaken to realize a fully circular strategy for concrete recycling. Yet, challenges such as diminished recycling efficiency, limited applicability across varying size fractions, the inability to recover cement paste, and economic infeasibility significantly hinder the ultimate aspiration of achieving a closed-loop concrete production and recycling process.

**Table S15** Overview of carbonation technology on concrete waste recycling

| Tech. **^$^**,  Ref,  Year | Mat. **^&^** [size, mm] | Oxide component **^§^** | Temp.  [°C] | Time  [h] | Key experimental conditions | Max C.D.,  CcP R.  or C.U. # | Key products (reutilization) | Recycling waste experimental material |
| --- | --- | --- | --- | --- | --- | --- | --- | --- |
| Wet C ^5^, 2025 | Arti.-D-P  [<0.15] | CaO: 61.4**^¥^**  SiO_2_: 19.0**^¥^**  Al_2_O_3_: 1.5**^¥^**  Fe_2_O_3_: 4.0**^¥^**  MgO: 2.2**^¥^**  Others: 11.9**^¥^** | Step I: 25;  Step II: 25, 80 | Step I:  0.5-1.5;  Step II: 0.33-2.33 | Step I:  a) 180 NL pure CO_2_/h;  b) 15-45% monoethanol-amine (MEA), 0.5 L;  Step II:  a) 50 g D-P;  b) 180 NL pure CO_2_/h; | C.D.:  About 80% | Cc and silica gel  (As SCM, 30% dosage in paste, comparable strength to the control batch) | MEA solution is expected to be recycled. |
| Wet C ^6^, 2024 | Real-D-S  [<0.4] | CaO: 16.9  SiO_2_: 58.6  Al_2_O_3_: 4.2  Fe_2_O_3_: 1.3  MgO: 1.1  Others: 17.9 | - | 4 | a) 300-500 g D-S;  b) 80 L/h synthetic air (23% CO_2_);  c) 0.2 and 1.0 wt% triethanolamine; | C.D.: 21% (increment) | Cc  (-) | - |
| Wet C ^7^,  2024 | Real-D-A  [4.75-9.5] | - | 23 | 1-2 | a) 30 L pure CO_2_/h;  b) L/S = 10; | C. U.:  5.26% | Coarse aggregate  (As aggregate, 100% dosage in concrete, superior strength to the uncarbonated batch) | - |
| Wet C ^8^, 2020 | Arti.-D-P  [<0.3] | CaO: 62.4**^¥^**  SiO_2_: 20.3**^¥^**  Al_2_O_3_: 5.27**^¥^**  Fe_2_O_3_: 2.4**^¥^**  MgO: 1.4**^¥^**  Others: 8.23**^¥^** | 20 | 0-2 | a) 10 L gas (CO_2_ concentration of 5-100%) /h/g cement; | C.D.: About 85% | Cc and Alumina-silica gel coexisted  (-) | - |
| Wet C ^9^, 2020 |  |  |  | 0-6 | a) 15g D-P;  b) 0.1 M NaOH, 0.15 L;  c) 10 L gas (CO_2_ concentration of 0-10%) /h/g cement; | C.D.: 100% |  | - |
| Wet C-Sonication ^10^, 2021 | Real-D-S  [0.15-0.6] | CaO: 27.75  SiO_2_: 50.85  Al_2_O_3_: 8.48  Fe_2_O_3_: 7.63  MgO: 3.69  Others: 1.6 | 50 | 0-8 | a) 10 g D-S;  b) 72 L CO_2_/h;  c) 250 W sonicator; | C.D.: 6% (increment) | Cc and silica gel coexisted  (-) | - |
| Wet C-Mechanical ^11^, 2024 | Real-D-P  [<0.2] | CaO: 44.35  SiO_2_: 27.09  Al_2_O_3_: 13.66  Fe_2_O_3_: 3.19  MgO: 2.07  Others: 9.64 | Room | 0.5 | a) 1.3 kg D-P;  b) 300-900 L/h pure CO_2_;  c) Pressure of 0.3 MPa;  d) Rotation of 300, 500, or 700 rpm; | C.D.: 67.1% | Cc & Silica gel coexisted  (As SCM, 0-100 wt% dosage to substitute fly ash in concrete, superior performance to control batch) | - |
| Chemical-  Wet C ^12^, 2023 | Arti.-D-P  [<0.075] | CaO: 71.11  SiO_2_: 16.51  Al_2_O_3_: 3.28  Fe_2_O_3_: 4.19  MgO: 0.95  Others: 3.96 | Step I: 25, 45, 65, 85; Step II: 15, 25, 50, 70 | Step I:0.083-2; Step II: 0.16-2 | Step I:  a) L/S=5-40;  b) 1-4 M NH_4_Cl solution, 0.2 L;  Step II:  a) CO_2_ pressure of 0.025-1 MPa;  b) 0.5-2 M NH_4_OH solution;  c) rotation of 500 rpm; | C. U.: about 27% | Cc & Calcium-silicate gel separated  (To be an accelerator in cement for early-age strength development) | Carbonation filtrate was utilized in four cycles. Leaching rate decreased from 65.7% to 20.5%. |
| Wet C ^13^, 2023 | Arti.-D-P  [<0.15] | CaO: 66.4**^¥^**  SiO_2_: 18.0**^¥^**  Al_2_O_3_: 5.5**^¥^**  Fe_2_O_3_: 4.0**^¥^**  MgO: 2.2**^¥^**  Others: 3.9**^¥^** | 25, 40, 80 | 0.083-1 | a) 50 g D-P;  b) 180 L/h or 300 L/h pure CO_2_; | C.D.: About 70% | Cc & Silica gel coexisted  (-) | - |
| Acc C ^13^, 2023 |  |  | - | 6, 18 | a) Pure CO_2_;  b) 0.2 MPa pressure; | C.D.: About 66% |  |  |
| Wet C ^14^,  2024 | Simu.-D-A  [5-10] | - | 25 | 0-2 | a) 12 L pure CO_2_/h/kg D-A;  b) Sea water;  c) L/S=10;  d) Rotation of 200 rpm; | - | Coarse aggregate with Cc formed on the surface  (As coarse aggregate, 100% dosage in concrete, lower strength than the natural aggregate batch) | - |
| Acc C ^14^,  2024 |  |  |  | 168 | CO_2_ concentration of 20%, RH= 65±5%; | - |  |  |
| Acc C ^15^,  2023 | Simu.-D-A  [5-20] | - | Room | 2, 4 | a) 4-6 kg D-A;  b) Pressure of 0.5/1/2 bar; | - | Coarse aggregate with Cc and silica gel coexisted on the surface  (As coarse aggregate, 100% dosage in concrete, superior strength to the uncarbonated batch) | - |
| Acc C ^16^, 2022 | Arti.-D-P  [<0.15] | CaO: 64.52  SiO_2_: 19.63  Al_2_O_3_: 5.43  Fe_2_O_3_: 3.21  MgO: 2.15  Others: 5.06 | 20, 60, 100, 140 | 0.5 | a) 10 g D-P, with 3g water;  b) 20% CO_2_ concentration;  c) Undefined pressure;  d) RH=5%-10%; | C.D.:  30.6% | Cc and Silica gel coexisted  (As SCM, 20% dosage in paste, comparable strength to pure cement paste) | - |
| Acc C ^17^, 2020 | Real-D-P  [<0.3] | CaO: 52.81  SiO_2_: 27.30  Al_2_O_3_: 8.79  Fe_2_O_3_: 3.83  MgO: 3.08  Others: 4.19 | 20 | 1-168 | a) 10 g D-P;  b) CO_2_ concentration of 20%;  c) Undefined pressure;  d) RH=65%;  e) L/S=0-0.7; | C. U.:  20.4% | Cc  (As SCM, 20% dosage in mortar, lower strength than the control batch) | - |
| Semi-dry C ^18^,  2023 | Simu.-D-P  [-] | CaO: 51.5  SiO_2_: 33.7  Al_2_O_3_: 4.6  Fe_2_O_3_: 2.8  MgO: 2.1  Others: 5.3 | 40-55 | 6 | a) 1 kg D-P;  b) 200 L pure CO_2_/h;  c) water-saturated gas; | C.D.:  74% | Cc and Alumina-silica gel coexisted  (As SCM, 20-40% dosage in mortar, superior strength to the control batch) | - |
| Wet C ^18^,  2023 |  |  | 30-45 |  | a) 1 kg D-P;  b) 200 L pure CO_2_/h;  c) L/S=3;  d) 0.05 M Na_2_SO_4_, 3L; | C.D.:  88% |  |  |
| Semi-dry C ^19^, 2022 | Arti.-D-P  [D_90_= 0.1/0.253] | - | 20 | 0-6 | a) 15 g D-P;  b) 50-100% RH;  c) 100 L gas (CO_2_ concentration of 5-25%) /h/g D-P; | CcP R.:  About 66 wt% | Cc and Alumina-silica gel coexisted  (-) | - |

(Note: **($)**: The carbonation technology is categorized into three types: wet carbonation in solution (Wet C), accelerated carbonation (Acc C), and carbonation with controlled moisture (Semi-dry C). **(&)**: The varied waste sample sizes and naming codes in the references made comparisons difficult and confusing, complicating data interpretation and analysis. To improve cross-sectional comparisons, we standardized the sample names according to the waste sample size used in this paper. The naming code in the table follows the format: A (the source of the waste sample) + B (the concrete waste within the specific size range). The sources of waste samples include artificial (Arti.) samples, simulated (Simu.) samples, and real (Real) samples. The artificial samples are the fabricated paste wastes using OPC and water without sand and aggregate. The simulated samples consist of hydrated cement paste and spent aggregate. The real samples are sourced from the demolishing plants in the real world. The classification method of the sample sizes in this study is utilized to define the size range of the concrete wastes studied in the references, including D-P (<0.3 mm), D-S (0.3-2.36 mm), and D-A (>2.36 mm). **(§)**: The value of “others” in the oxide component was calculated by 100wt% minus the amount of the content of CaO+SiO_2_+Al_2_O_3_+Fe_2_O_3_+MgO due to some missed data, like LOI and the content of other metal oxides in the reference. **(¥)**: The oxide components of the waste used in the studies ^5, 8, 9, 13^ were not given. However, the waste was fabricated using only cement and water. Therefore, the oxide components of waste are assumed to be the same as the cement. **(#)**: Three parameters were normally used to evaluate the effectiveness or environmental impact of carbonation mineralization technology on concrete waste, reported in the references, including carbonation degree (C.D.), calcium carbonate precipitation rate (CcP R.), and CO_2_ uptake (C.U.). The explanation of these three parameters is as follows: (1) C.D.: the reaction extent to which CO_2_ has reacted with the sample, (2) CcP R.: the weight of newly precipitated calcium carbonate per unit weight of the sample, (3) C.U.: the weight of CO_2_ absorbed per unit weight of the sample. L/S: the weight of liquid to that of solid. RH: Relative humidity. **(-)**: Not mentioned in the study.)

**Table S16** Overview of advanced technologies for concrete waste recycling

| Tech.,  Ref,  Year | Mat. **^&^** [size, mm] | Oxide component **^§^** | Temp.  [°C] | Time (h) | Key experimental conditions | Products | Reutilization or potential application of products | Recycling waste experimental material |
| --- | --- | --- | --- | --- | --- | --- | --- | --- |
|  |  |  |  |  |  |  |  |  |
| Acid ^20^,  2022 | Real-D-A  [<4.75] | CaO: 12.47  SiO_2_: 63.36  Al_2_O_3_: 8.22  Fe_2_O_3_: 3.31  MgO: -  Others: 12.64 | 20, 40,  60, 80 | 0-8 | a) 50 g D-A;  b) 0.4-1 M acetic acid, 0.25-0.45 L;  c) L/S=5/7/9; | Ca^2+^ leachate, silica-rich residue, recycled sand | Recycled sand as fine aggregate in mortar, superior strength to standard sand batch;  Silica-rich residue could be SCM with pozzolanic reactivity | Acid and alkali could be potentially recycled. |
| Acid-  Thermal ^21^, 2019 | Real-D-A  [<10] | - | Step I: Room;  Step II:  400; | Step I: 24;  Step II: 0.67; | Step I:  a) 2 kg D-A;  b) 0.5-10 M acetic acid, 2 L;  Step II:  a) Muffle furnace; | Treated aggregate | As aggregate, 100% dosage in mortar,  superior strength to the untreated batch | - |
| Thermal  -Acid ^22^, 2017 | Real-D-A  [<4.75] | CaO: ~40  SiO_2_: ~50  Al_2_O_3_: <5  Fe_2_O_3_: <5  MgO: -  Others: - | Step I: 300, 400, 500, 600;  Step II:  Room; | Step I: 2;  Step II: 24; | Step I: Muffle furnace;  Step II: 0.1-0.7 M HCl; | Treated aggregate | As aggregate, 100% dosage in concrete,  lower strength than the natural aggregate batch | - |
| Acid-Pozzolanic slurry ^23^, 2016 | Real-D-A  [<20] | CaO: 18.84  SiO_2_: 53.44  Al_2_O_3_: 11.9  Fe_2_O_3_: 5.9  MgO: 0.94  Others: 8.98 | Room | Step I: 24;  Step II: 24; | Step I:  10% normality HCl/HNO_3_/  H_2_SO_4_;  Step II: Silica fume coating; | Treated aggregate | As aggregate, 100% dosage in concrete, lower strength than the natural aggregate batch | - |
| Acid ^24^,  2024 | Simu.-D-A  [4-22.4] | - | 22 | 24 | 0.3-3 M HCl/H_2_SO_4_; | Treated aggregate | As aggregate, 100% dosage in concrete,  lower strength than the natural aggregate batch | - |
| Acid-Mechanical ^25^, 2024 | Real-D-A  [4.75-20] | - | Room | Step I: 168;  Step II:  0.16 | Step I: 5% acetic acid;  Step II: Mechanical rubbing; | Treated aggregate | As aggregate, 0-100% dosage in concrete, lower strength than the natural aggregate batch | - |
| Acid-Mechanical ^26^, 2024 | Real-D-A  [4.75-40] | - | Room | Step I: 24;  Step II:  0-0.167; | Step I:  0.5 M HCl;  Step II: Abrasion treatment, 25rpm; | Treated aggregate | As aggregate, 0-100% dosage in concrete, superior strength to the natural aggregate batch | - |
| Acid-Mechanical ^27^, 2022 | Real-D-A  [4.75-25] | - | Room | Step I: 72;  Step II:  0-0.2; | Step I:  a) 10 kg D-A;  b) 3% acetic acid;  Step II:  a) Grinding at 33 rpm with 10 iron balls; | Treated aggregate | As aggregate, 100% dosage in concrete,  lower strength than the natural aggregate batch | - |
| Acid ^28^,  2024 | Real-D-S  [0.3-0.6] | CaO: ~52  SiO_2_: ~16  Al_2_O_3_: ~3  Fe_2_O_3_: ~28  MgO: -  Others: ~1 | Room | 24 | 0.1-0.7 M HCl; | Treated aggregate | As aggregate, 30% dosage in concrete, superior strength to the natural aggregate batch | - |
| Acid ^29^ | Real-D-A  [4.75-20] | - | - | 24 | 0.8 M HCl; | Treated aggregate | As aggregate, 100% dosage in concrete, comparable strength to the natural aggregate batch | - |
| Thermal ^29^ |  |  | 1500 | 24 | Muffle furnace; |  |  |  |
| Water washing ^29^, 2022 |  |  | - | 0.25-0.33 | Pressure washing; |  |  |  |
| Acid-Mechanical ^30^ | Real-D-A  [4.75-25] | - | Room | Step I: 24;  Step II:  0.25 | a) 0.1 M HCl & acetic acid;  b) Mechanical treatment; | Treated aggregate | - | - |
| Thermo-Mechanical ^30^, 2016 |  |  | 250, 350, 500, 750 | Step I: 1;  Step II:  0.25 | a) Muffle furnace;  b) Mechanical treatment; |  |  |  |
| Mechanical ^31^, 2019 | Real-D-A  [4-22.4] | - | - | - | Ball milling,  100-500 drum rotation and 0-12 steel balls; | Treated aggregate | As aggregate, 60% dosage in concrete, comparable strength to the natural aggregate batch | - |
| Mechanical ^32^, 2022 | Real-D-P  [<1] | CaO: 31.713  SiO_2_: 29.689  Al_2_O_3_: 7.948  Fe_2_O_3_: 2.453  MgO: 2.728  Others: 25.469 | - | - | a) Grinding machine, treatment capacity of 0.6-3 t/h or 4-48 kg/h;  b) L/S=1; | Fine powder | As SCM, 0-15% dosage in concrete, comparable strength to the natural aggregate batch | - |
| Thermo-Mechanical ^33^, 2021 | Real-D-A  [<50] | - | Step I:  300-700;  Step II: - | Step I:  0.5-2;  Step II:  0.25; | Step I: Muffle furnace;  Step II: Ball milling, 33 rpm; | Treated aggregate | As aggregate, 100% dosage in concrete, lower strength than the natural aggregate batch | - |
| Thermo-Mechanical ^34^, 2023 | Real-D-A  [20] | - | Step I:  300-800;  Step II: - | Step I:  4;  Step II: - | Step I: Muffle furnace;  Step II: Abrasion treatment, 33 rpm; | Treated aggregate | As aggregate, 100% dosage in concrete, lower strength than the natural aggregate batch | - |
| Thermo-Mechanical ^35^, 2021 | Real-D-A  [4.75-13] | - | Step I:  600;  Step II: - | Step I:  1;  Step II: 1-12 | Step I: Muffle furnace;  Step II: Milling machine, 140 rpm; | Treated aggregate | - | - |
| Pozzolanic slurry ^36^, 2018 | Real-D-A  [12.5] | CaO: 12.01  SiO_2_: 62.56  Al_2_O_3_: 12.52  Fe_2_O_3_: 5.82  MgO: 1.83  Others: 5.26 | 20 | Step I: 1;  Step II: 4; | Step I: 10% Na_2_SiO_3_;  Step II: 3-7 wt% silica fume; | Treated aggregate | As aggregate, 100% dosage in concrete, lower strength than the natural aggregate batch | - |
| Pozzolanic slurry ^37^, 2021 | Real-D-A  [4.75-25] | - | 25 | 0.5 | a) 20-50% cement-silica fume slurry;  b) 20-50% Na_2_SiO_3_; | Treated aggregate | As aggregate, 0-100% dosage in concrete, lower strength than the natural aggregate batch | - |
| Pozzolanic slurry ^38^, 2023 | Real-D-A  [4.75-9.5] | - | Room | 48 | a) 0-2.5% nano-silica suspension;  b) Normal pressure or 1 bar; | Treated aggregate | As aggregate, 100% dosage in concrete, comparable strength to the natural aggregate batch | - |
| Pozzolanic slurry ^39^, 2024 | Real-D-A  [<19.5] | - | - | 12 or 24 | Nano-silica, micro zeolite, and nano montmorillonite; | Treated aggregate | As aggregate, 100% dosage in concrete, comparable strength to the natural aggregate batch | - |
| Pozzolanic slurry ^40^, 2024 | Real-D-A  [<25] | - | - | 24 | 2-30% nano-silica solution; | Treated aggregate | As aggregate, 100% dosage in concrete, superior strength to the untreated batch | - |
| Pozzolanic slurry ^41^, 2023 | Simu.-D-A  [5-20] | - | Room | 24, 48, 72 | 0-3% nano-silica solution; | Treated aggregate | As aggregate, 50-100% dosage in concrete, lower strength than the natural aggregate batch | - |
| Pozzolanic slurry ^42^, 2023 | Simu.-D-A  [5-20] | - | Room | 24 | 2% nano-silica mixed with 15% Na_2_SiO_3_; | Treated aggregate | As aggregate, 50-100% dosage in concrete, lower strength than the natural aggregate batch | - |
| Polymer impregnation ^43^, 2017 | Real-D-A  [<9.52] | - | - | 24 | a) 1-2 kg D-A;  b) 20% silane solution; | Hydro-phobic aggregate | - | - |
| Polymer impregnation ^44^, 2023 | Real-D-A  [5-30] | - | - | 12-24 | F0704 aqueous epoxy resin; | Treated aggregate | As aggregate, 100% dosage in concrete, comparable strength to the natural aggregate batch | - |
| Polymer impregnation ^45^, 2021 | Real-D-A  [5-10] | - | 23 | 36 | 10% methyl triethoxysilane; | Treated aggregate | As aggregate, 100% dosage in concrete, comparable strength to the untreated batch | - |
| Polymer impregnation ^46^, 2019 | Real-D-A  [5-10] | - | Room | 24 | 10% methyl triethoxysilane and poly (dimethylsiloxane); | Treated aggregate | As aggregate, 100% dosage in concrete, superior strength to the untreated batch | - |
| Polymer impregnation ^47^, 2023 | Real-D-A  [5-25] | - | 23 | - | 0.05% polymer solution; | Treated aggregate | As aggregate, 100% dosage in concrete, lower strength to the natural aggregate batch | - |
| Polymer impregnation ^48^, 2021 | Real-D-A  [12-22] | CaO: ~13  SiO_2_, Al_2_O_3_, Fe_2_O_3_: >65  MgO: -  Others: - | - | 0-1 | 10-20% acrylic resin; | Treated aggregate | As aggregate, 100% dosage in concrete, comparable strength to the natural aggregate batch | - |
| Polymer impregnation, ^49^, 2022 |  |  | 20 | 1 | 20% acrylic resin; |  | As aggregate, 100% dosage in concrete, comparable strength to the untreated batch | - |
| Bio deposition ^50^, 2023 | Real-D-A  [10-20] | CaO: 14.17  SiO_2_: 43.35  Al_2_O_3_: 14.60  Fe_2_O_3_: 1.4  MgO: 8.23  Others: 18.25 | Room | 84 | 10^8^-10^9^ CFU/mL bacteria, 0.4 wt% sodium alginate solution, 0.25-1 M urea solution; | Treated aggregate | As aggregate, 100% dosage in concrete, lower strength than the natural aggregate batch | - |
| Bio deposition ^51^, 2018 | Real-D-A  [<20] | - | 26 | 480 | 10^8^ cell/ml bacteria solution, culture solution containing natrium lacticum (7.5 ml/L), calcium chloride (3.33 g/L), and CAPS (22.13 g/L); | Treated aggregate | As aggregate, 100% dosage in mortar, lower strength than the natural aggregate batch | - |
| Bio deposition ^52^, 2017 | Real-D-A  [-] | - | 20 | 96 | 3 × 10^8^ cell/ml Bacillus sphaericus LMG 22257,  sterile medium consisting of yeast extract (20 g/L) and urea (20 g/L), deposition medium consisting of urea (0.5 M) and Ca-nitrate (0.5 M); | Treated aggregate | As aggregate, 100% dosage in concrete, superior strength to the natural aggregate batch | - |
| Microwave ^53^, 2024 | Arti.-D-P  [<0.075] | CaO: 45.40  SiO_2_: 23.52  Al_2_O_3_: 10.84  Fe_2_O_3_: 4.23  MgO: -  Others: 16.01 | - | 0.083-0.25 | Microwave power: 210, 490, 700 W; | Activated powder | As SCM, 30% dosage in mortar, lower strength than the control batch | - |
| Microwave ^54^, 2024 | Arti.-D-P  [<0.16] | CaO: 47.58  SiO_2_: 17.72  Al_2_O_3_: 5.17  Fe_2_O_3_: 2.75  MgO: 2.81  Others: 23.97 | - | 2 | Microwave powers: 300, 500, 800 W; | Activated powder | As SCM, 30% dosage in mortar, lower strength than the control batch | - |

(Note: **(&)**: the sample names according to the waste sample size used in this paper are standardized as in the annotation in Table S15. **(§)**: The value of “others” in the oxide component was calculated by 100wt% minus the amount of the content of CaO+SiO_2_+Al_2_O_3_+Fe_2_O_3_+MgO due to some missed data, like LOI and the content of other metal oxides in the reference. **(-)**: Not mentioned in the study.)

**Supplementary Appendix B**: Mixture design of reversed Portland clinker

The raw feed design of r-Pcl, exclusively using hydroxide precipitation and silica precipitation, was developed by the following procedures. The ratio of these two materials was calculated by considering three key parameters that control burnability and ensure effective clinkering, including lime saturation factor (LSF), silica modulus (SM), and aluminium ratio (AR), as follows. These parameters are widely applied in commercial OPC fabrication. The common range is 92-98 for $LSF$, 2.1-2.7 for $SM$, and 1.0-2.5 for $AR$ ^55^, respectively.

|  | $LSF=\frac{100 w\left( CaO \right)}{2.8w\left( SiO_{2} \right)+1.18w\left( {Al}_{2}O_{3} \right)+0.65w\left( {Fe}_{2}O_{3} \right)}$ | (1) |
| --- | --- | --- |
|  | $SM=\frac{w\left( SiO_{2} \right)}{w\left( {Al}_{2}O_{3} \right)+w\left( {Fe}_{2}O_{3} \right)}$ | (2) |
|  | $AR=\frac{w\left( {Al}_{2}O_{3} \right)}{w\left( {Fe}_{2}O_{3} \right)}$ | (3) |

Where $w\left( CaO \right)$, $w\left( SiO_{2} \right)$, $w\left( {Al}_{2}O_{3} \right)$, and $w\left( {Fe}_{2}O_{3} \right)$ is the normalized weight percentage in feedstock, respectively.

Since there are only two raw feeds (hydroxide precipitation and silica precipitation), changing the ratio of them will simultaneously change the values of these three parameters. In this work, alite is one of the main target mineral phases. Therefore, LSF is the priority in three parameters because it directly determines the content of alite in r-Pcl. According to the oxide components and LOI of the raw materials listed in Table S2, the final mass ratio of hydroxide precipitation and silica precipitation in the raw feed was 88.15:11.85, leading to LSF of 99.48, SM of 2.04, and AR of 0.84.

For the case study, these parameters were designed again according to oxide components and LOI of hydroxide(HK) and silica(HK) in Table S11, as LSF: 98.31, SM: 1.57, and AR: 1.19. Accordingly, the final mass ratio of hydroxide(HK) and silica(HK) was 92.79:7.21.

**Supplementary Appendix C**: Environmental and economic assessment of natural resources-based and electrochemical-driven cement and concrete

**1. Life cycle assessment**

**Goal and scope:**

The environmental impact was assessed utilizing the Life Cycle Assessment (LCA) methodology as outlined in ISO 14040 ^56^ and ISO 14044 ^57^. Cement-making was evaluated from a cradle to gate perspective, while concrete was assessed from a cradle to grave perspective, covering the entire life cycle from manufacturing and application to end-of-life disposal. The primary goal was to comparatively evaluate the global warming potential (GWP100) of systems based on natural resources and waste-derived feedstocks, to find out the potential strategies for establishing sustainable and circular concrete material systems.

**Functional unit:**

Two functional units were defined for this study: 1 t of cement (for the cradle to gate assessment of cement-making) and 1 m^3^ of concrete (for the cradle to grave assessment of concrete). These units provide a consistent basis for comparing environmental impacts across different material formulations and life cycle stages.

**System boundary:**

This study employed a cradle to gate system boundary for cement-making and a cradle to grave system boundary for concrete, to assess both conventional and electrochemical-driven cement and concrete systems (Figure S9).

For conventional OPC production (OPC-0%), the boundary includes upstream raw material extraction (limestone, clay, sand, and iron ore), material processing (production, transportation, grinding, and mixing), clinker calcination, and cement preparation. After calcination, 5 wt% gypsum is added to the clinker to regulate the setting time, and the mixture is then ground to prepare the final OPC product. A blended cement variant (OPC-50%), commonly known as LC^3^, was also considered, consisting of 50 wt% OPC clinker, 30 wt% calcined clay, 15 wt% limestone, and 5 wt% gypsum.

In contrast to traditional cement-making manufacturing, the RPC(HK) is prepared almost entirely from electrochemically treated wastes. The concrete waste is transported to the processing facility, where it is crushed, sieved, and then subjected to the ER process. This yields two key products: hydroxide(HK) and silica(HK), which are subsequently used as the exclusive feedstocks for making r-Pcl(HK). As a result, the conventional upstream inputs, limestone, clay, sand, and iron ore, are entirely avoided. The same formulation and cement manufacturing process were adopted to produce both the pure RPC (RPC-0%) and the blended variant (RPC-50%), the latter mirroring the composition of LC^3^ by incorporating calcined clay and limestone.

During clinker calcination, thermal energy is supplied by fossil fuels. The cement industry has traditionally relied on a range of fossil fuels, including coal, oil, petroleum coke, and natural gas, for kiln and pre-heater operations ^58, 59^. To reflect this practice while maintaining comparability across systems, a 50:50 mixture of coal and natural gas as an energy supply was adopted in this study, both of which are well-documented fossil fuels with standardized life cycle inventory data. Coal-gas co-firing has been increasingly adopted in the cement industry as a transitional strategy to reduce thermal carbon intensity ^60, 61^. Industrial cement plants operating under coal-gas co-firing at 50:50 energy shares have been reported ^62^. Accordingly, this mixed-fuel scenario reflects a realistic practice rather than an idealized assumption.

The life cycle of concrete was assessed under a cradle to grave boundary, subdivided into three stages: cradle to gate, gate to gate, and gate to grave.

(1) Cradle to gate. For traditional concrete manufacturing (N-C), the input materials include OPC, natural sand, natural aggregates, and water. After transportation to a ready-mix plant, these materials are batched and mixed according to a predefined proportion (Table S8) to produce fresh concrete. For the reversed concrete (RC) system, RPC is used as the binder, while the aggregate is sourced from electrochemical-recycled granules with various size fractions, including ER-P(HK), ER-S(HK), and ER-A(HK). The final RC(HK) mix was manufactured using RPC(HK), ER-S(HK), ER-A(HK), and water, following the same mix proportion (Table S8) to ensure comparability. Substances, including ER-P(HK), surplus ER-S(HK), surplus ER-A(HK), and remaining silica(HK), were collected as a group of Remains (Figure 5C). To enable consistent comparison, both pure cements (OPC-0% or RPC-0%) and their blended variants (OPC-50% and RPC-50%) were modelled.

(2) Gate to gate. The in-use stage incorporated natural carbonation of concrete, reflecting whole-life carbon dynamics. Concrete is widely recognized as a passive carbon sink, with estimates suggesting that carbonation offset approximately 43% of historical CO_2_ emissions from cement production between 1930 and 2013 ^63^. According to published studies ^64, 65^, the extent of in-use carbonation is closely correlated with clinker content, which determines the quantity of carbonatable phases (e.g., portlandite and calcium silicate hydrates). Following conservative estimates, N-C made with OPC-0% was assumed to reabsorb 30% of its cradle to gate embodied carbon of cement-making, while OPC-50% concrete was assigned a 15% uptake. The same reabsorption rates were applied to RC concretes, owing to their identical concrete mix proportions and comparable clinker composition.

(3) Gate to grave. End-of-life scenarios were distinguished between the two systems. For the N-C system, disposal was modelled as landfilling in inert sites. Secondary carbonation of residual hydrates during landfilling was not considered, as its extent is time- and condition-dependent, and critically, to ensure comparability with the ER scenario, where concrete is directly recycled without an interim storage phase. Only CO_2_ emissions from waste transportation and on-site management before landfilling were included. In contrast, aged RC was modelled as being fully reintegrated into the ER process at the end of life, thereby avoiding landfilling and completing the recycling loop.


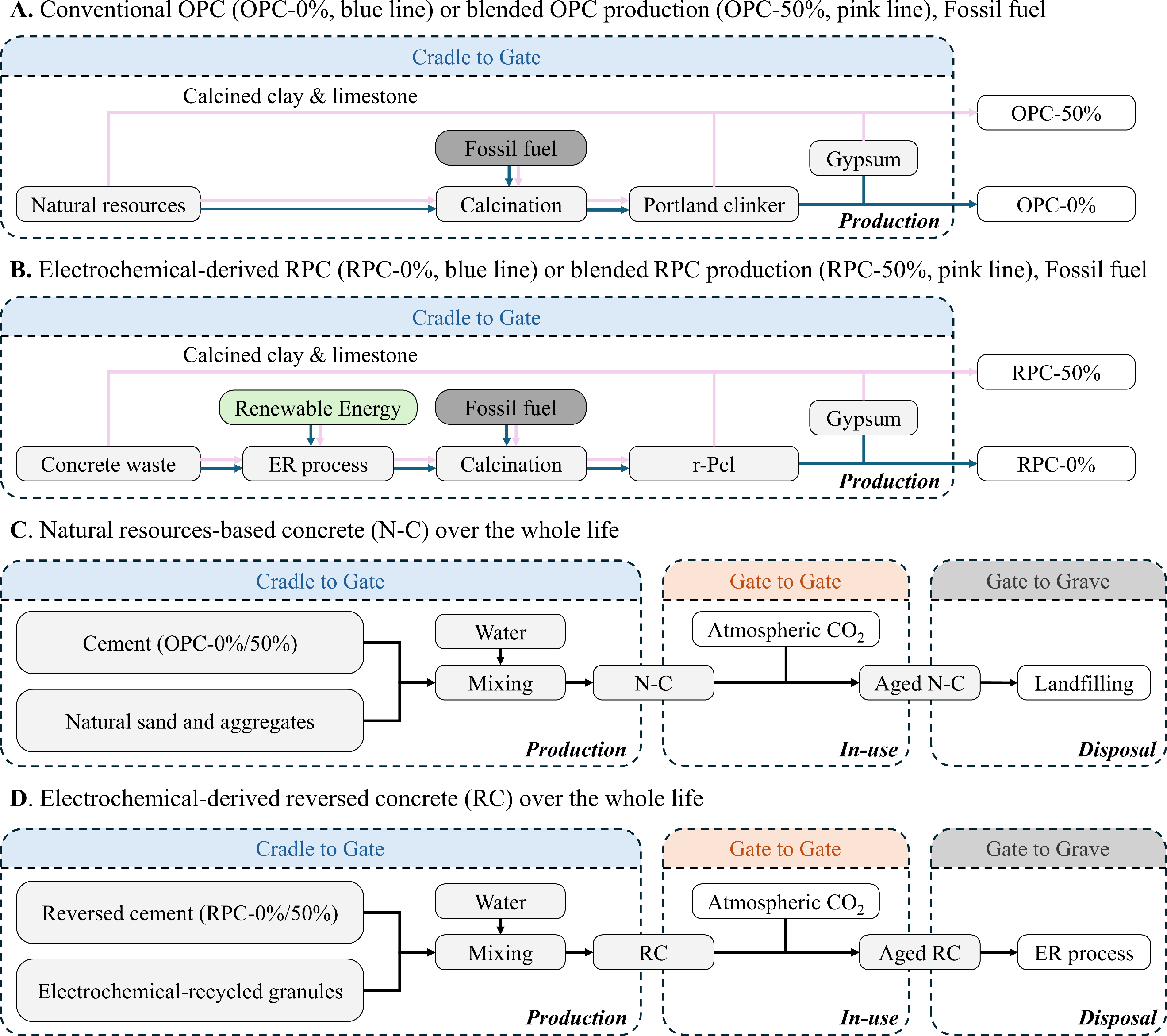


**Figure S9** **System boundaries. (A)** Conventional OPC or blended OPC production; (**B**) Electrochemical-derived RPC or blended RPC production; (**C**) Natural resources-based concrete (N-C) over the whole life; (**D**) Electrochemical-derived reversed concrete (RC) over the whole life.

**Inventory analysis and life cycle impact assessment:**

The inventory analysis entails quantifying all inputs and outputs associated with a product over its entire life cycle. The impacts were assessed by OpenLCA software using the IPCC 2013 method, which is a widely adopted assessment methodology for environmental impacts. The primary impact damage category pertinent to cement and concrete, namely GWP100, was chosen ^66^. The raw material compositions of OPC clinker were defined based on commercially available cement types produced in HK ^67^, listed in Table S17. To maintain consistency, no limestone powder was added to the cement. Therefore, the composition of OPC cement analysed in the LCA contained 95 wt% clinker and 5 wt% gypsum. To conduct comprehensive LCA assessments, a complete dataset summarized in Table S18 and Table S19 was gathered from the literature and Ecoinvent database. For electrochemical-driven cement and concrete manufacturing, the electricity input is assumed to be supplied by renewable energy sources, specifically run-of-river hydropower, which is a technically mature solution ^68^ and represents a realistic near-future scenario. The assumption of using renewable energy is consistent with previous electrochemical-related studies ^69, 70^.

**Table S17.** Raw material compositions for producing 1 t of OPC clinker [t].

| Limestone | Clay | Sand | Iron ore |
| --- | --- | --- | --- |
| 1.23 | 0.14 | 0.14 | 0.03 |

(Note: Assuming CO_2_ emission of calcinating 1 t limestone equals 0.44 t and other raw materials do not emit CO_2_ during the calcination. Therefore, the total CO_2_ emission for producing 1 t OPC clinker from raw material decomposition corresponds to 0.541 t.)

**Table S18.** Energy requirements of materials and wastes in processing and transportation.

| Process | Energy consumption |
| --- | --- |
| Limestone production ^a)^ | D |
| Clay production ^a)^ | D |
| Iron ore production ^a)^ | D |
| Sand production ^a)^ | D |
| Gypsum production | D |
| Coal production ^a)^ | D |
| Coal heating value | 23.9 GJ/t ^71^ |
| Natural gas heating value | 47.1 MJ/kg ^69^ or 36.6 MJ/m^3^ ^72^ |
| CO_2_ emission factor of hard coal | 93.9 kg/ GJ ^73^ |
| CO_2_ emission factor of natural gas | 56.1 kg/GJ ^69^ |
| Electricity consumption (crushing, raw materials grinding, and clinker production) | 87 kWh/t clinker ^67^ |
| Coal combustion | D |
| Cement mixing | 48 kWh/t cement ^67^ |
| Natural Sand production  (Extracting, screening, crushing, and sieving) | 27 MJ/t (electricity)  & 50 MJ/t (diesel) ^74^ |
| Natural aggregate production  (Extracting, screening, crushing, and sieving) | 22 MJ/t (electricity)  & 51 MJ/t (diesel) ^74^ |
| On-site concrete waste management  (Hydraulic breaking hammer) | 0.0963 kg/m^3^ (diesel) ^75^  (Heating value of 44 MJ/kg) ^71^ |
| Spent aggregate production  (Crushing and screening) | 0.515 kWh/t ^75^ |
| Washing, drying, and shaking | 3.44 kWh/t ^b)^ ^76^ |
| Concrete mixing | 0.458 kWh/m^3^ concrete ^77^ |
| Electricity consumption  (Waste treatment via ER strategy) | 0.471 kWh/kg waste ^c)^ |
| Transport, sea (Inland barge) | D |
| Transport, sea (Transoceanic ship) | D |
| Transport, road (Trucks, 7.5-16 t) | D |
| Transport, road (Trucks, >32 t) | D |
| Grid emission factor | D |

(Note: D: The value of energy consumption for the principal processes is directly extracted from the database, excluding infrastructure. ^a)^ Raw material preparations include quarrying and processing. ^b)^ The data was referred to a similar study that produced hydrated lime using CaCO_3_, including washing machine operation (0.78 kWh/t powder), drying machine operation (1.33 kWh/t powder). The rest electricity of 1.33 kWh/t powder was attributed to the shaking machine operation, which was assumed to have the same energy consumption as the drying machine operation. ^c)^ The electricity consumption of each unit operation was directly measured during pilot-scale operation using in situ energy monitoring of the corresponding equipment. The recorded electricity consumption (kWh) for each operation was normalized to the mass of concrete waste processed in each batch to obtain the electricity demand per unit mass (kWh/kg concrete waste). All reported values correspond to representative pilot-scale operation and form the basis for the LCA and economic assessments presented in Section 2.5.

**Table S19.** Transportation data of raw materials and wastes.

| Materials | Locations | Transport type | Distance |
| --- | --- | --- | --- |
| Limestone | Guangdong, China  to Hong Kong Port | Inland barge | 250 km ^78^ |
| Clay | Guangdong, China  to Hong Kong Port | Inland barge | 250 km ^78^ |
| Sand | Guangdong, China  to Hong Kong Port | Inland barge | 250 km ^78^ |
| Iron ore | South Japan to Hong Kong Port | Transoceanic ship | 3202 km ^78^ |
| Coal | Indonesia to Hong Kong Port | Transoceanic ship | 4073 km ^67^ |
| Gypsum | Local (Power Plant) | Trucks (7.5-16 t) | 7 km ^67^ |
| Cement | Local | Trucks (>32 t) | 30 km ^a)^ ^78^ |
| River sand | Extraction sites to Dongguan, Guangdong Province, China | Trucks (>32 t) | 20 km ^74^ |
|  | Dongguan to Hong Kong Port | Inland barge | 128 km ^74^ |
|  | Hong Kong Port to utilization sites | Trucks (>32 t) | 30 km ^74^ |
| Natural aggregates | Extraction sites to Dongguan, Guangdong Province, China | Trucks (>32 t) | 50 km ^74^ |
|  | Dongguan to Hong Kong Port | Inland barge | 128 km ^74^ |
|  | Hong Kong Port to utilization sites | Trucks (>32 t) | 30 km ^74^ |
| Demolished concrete waste | Concrete waste from sites to the processing plant | Trucks (>32 t) | 20 km ^79^ |
| Electrochemical- recycled granules | Processing plant to utilization sites | Trucks (>32 t) | 20 km ^b)^ |
| Landfilling | Concrete waste from sites to  landfill site | Trucks (>32 t) | 20 km ^b)^ |

(Notes: ^a)^ According to the study that gave the local distance ranging from 3-48 km ^78^, the transportation distance of cement used in Hong Kong is set as 30 km in this study. ^b)^ This value refers to the transportation distance of concrete waste from sites to the processing plant.)

**2. Economic evaluation**

To compare the costs of the conventional and electrochemical routes for concrete, the parameters used in the net operating cost analysis are summarized in Table S20. This analysis considers process-related expenditures, specifically electricity consumption, kiln fuel usage, raw material inputs, and carbon pricing. End-of-life waste management is treated differently for the two systems: in the N-C pathway, landfill disposal costs are included as an expense based on regionally variable tipping fees, whereas in the ER pathway, the avoided landfill cost is credited to reflect the economic value of diverting concrete waste from disposal through electrochemical valorisation. A full techno-economic assessment is not attempted, as key factors such as capital investment, system performance, and equipment lifespan remain undefined at an industrial scale. Instead, this analysis provides a focused estimate of the operational economic feasibility of the ER pathway. For RC, six scenarios are evaluated by combining three electricity price levels (optimistic, realistic, pessimistic) with two landfill fee levels (low and high), representing possible variations in energy costs and regional waste management policies. For N-C, two scenarios are considered as the reference batches, both using realistic electricity prices but differing in landfill fee levels (low and high).

**Table S20.** Parameters for net operating cost analysis of conventional concrete and ER-based concrete

|  | Electricity cost ($/kWh) | Coal cost  ($/t) | Natural gas cost ($/kWh) | Carbon price ($/t) | Feedstock cost for clinkering ($/t cement) | | Aggregate cost  ($/t) | Landfill fee ($/t) |
| --- | --- | --- | --- | --- | --- | --- | --- | --- |
|  |  |  |  |  | Limestone | Others |  |  |
| RC(opti, low) | 0.023 | 140 | 0.076 | 32 | - | - | - | 0.89 |
| RC(real, low) | 0.05 | 140 | 0.076 | 32 | - | - | - | 0.89 |
| RC(pess, low) | 0.1 | 140 | 0.076 | 32 | - | - | - | 0.89 |
| RC(opti, high) | 0.023 | 140 | 0.076 | 32 | - | - | - | 50 |
| RC(real, high) | 0.05 | 140 | 0.076 | 32 | - | - | - | 50 |
| RC(pess, high) | 0.1 | 140 | 0.076 | 32 | - | - | - | 50 |
| N-C(real, low) | 0.05 | 140 | 0.076 | 32 | 40 | 20 | 13.9 | 0.89 |
| N-C(real, high) | 0.05 | 140 | 0.076 | 32 | 40 | 20 | 13.9 | 50 |

(Note: Three electricity costs represent different market conditions: optimistic ($ 0.023/kWh), realistic ($ 0.05/kWh), and pessimistic ($ 0.1/kWh), denoted as opti, real, and pess, respectively. Two landfill fees reflect regional policy variations: low ($ 0.89/t) and high ($ 50/t), denoted as low and high, respectively. Data sources: Electricity cost ^80, 81^; Coal cost (varies with region to region, thus taking an assumption of the average value of 140$/ton, ^82^); Natural gas cost (Global average, ^83^); Energy requirement for conventional cement process (3.3 GJ/t, ^84, 85^); Carbon price (Global average, ^86, 87^); Feedstock cost for clinkering ^88^; Aggregate cost ^89^; Landfill fee (varies by region, ^90-92^).)

**Supplementary Appendix D**: Resource consumption for concrete fabrication

To fabricate 1 m^3^ N-C, the mixture requires 410 kg of OPC, 636 kg of sand, and 1131 kg of aggregate, as specified in Table S8. The 410 kg of OPC consists of 389.5 kg of Portland clinker and 20.5 kg of gypsum. According to Table S17, fabricating 389.5 kg of Portland clinker requires 479.09 kg of limestone, 54.53 kg of clay, 54.53 kg of sand, and 11.69 kg of iron ore. In total, producing 1 m^3^ N-C consumes 2366.84 kg (rounded to 2367 kg) of natural resources.

To fabricate 1 m^3^ RC(HK), the case study serves as the reference. The mixture requires 410 kg of RPC(HK), 636 kg of ER-S(HK), and 1131 kg of ER-A(HK), as detailed in Table S8. The 410 kg of RPC(HK) is composed of 95 wt% (389.5 kg) of r-Pcl(HK) and 5 wt% (20.5 kg) of gypsum. Based on the weight ratio of each recycled substance and feedstock design (Table S12 and Supplementary Appendix B), producing 389.5 kg of r-Pcl(HK) requires 2925.27 kg (rounded to 2925 kg) of real concrete waste from HK. After electrochemical processing, 705.77 kg of ER-S(HK) and 1228.87 kg of ER-A(HK) can be recovered, fulfilling the mixture requirements for 1 m^3^ RC(HK). In summary, fabricating 1 m^3^ RC(HK) requires 2925 kg of real concrete waste from HK.

**SI References**

(1) Silva, D. A.; Roman, H. R.; Gleize, P. J. P. Evidences of chemical interaction between EVA and hydrating Portland cement. *Cem. Concr. Res.* **2002**, *32* (9), 1383-1390. DOI: <https://doi.org/10.1016/S0008-8846(02)00805-0>.

(2) Lyu, H.; Hao, L.; Zhang, S.; Poon, C. S. High-performance belite rich eco-cement synthesized from solid wastes: Raw feed design, sintering temperature optimization, and property analysis. *Resour. Conserv. Recy.* **2023**, *199*, 107211. DOI: <https://doi.org/10.1016/j.resconrec.2023.107211>.

(3) Driver, J. G.; Bernard, E.; Patrizio, P.; Fennell, P. S.; Scrivener, K.; Myers, R. J. Global decarbonization potential of CO<sub>2</sub> mineralization in concrete materials. *PNAS* **2024**, *121* (29), e2313475121. DOI: doi:10.1073/pnas.2313475121.

(4) Poon, C. S.; Shen, P.; Jiang, Y.; Ma, Z.; Xuan, D. Total recycling of concrete waste using accelerated carbonation: A review. *Cem. Concr. Res.* **2023**, *173*, 107284. DOI: <https://doi.org/10.1016/j.cemconres.2023.107284>.

(5) Mao, Y.; Hu, X.; Drissi, S.; Chen, W.; Shi, C. Wet carbonation of recycled cement paste powder using a CO2-loaded monoethanolamine solvent as an internal CO2 source. *Resour. Conserv. Recy.* **2025**, *212*, 107901. DOI: <https://doi.org/10.1016/j.resconrec.2024.107901>.

(6) Teune, I. E.; Schollbach, K. Triethanolamine-promoted separation of calcium from recycled concrete fines during aqueous carbonation. *Resour. Conserv. Recy.* **2024**, *206*, 107604. DOI: <https://doi.org/10.1016/j.resconrec.2024.107604>.

(7) Shuvo, A. K.; Sarker, P. K.; Shaikh, F. U. A.; Rajayogan, V. Improvement of crushed returned concrete aggregates by wet carbonation. *Constr. Build. Mater.* **2024**, *448*, 138253. DOI: <https://doi.org/10.1016/j.conbuildmat.2024.138253>.

(8) Zajac, M.; Skibsted, J.; Durdzinski, P.; Bullerjahn, F.; Skocek, J.; Ben Haha, M. Kinetics of enforced carbonation of cement paste. *Cem. Concr. Res.* **2020**, *131*, 106013. DOI: <https://doi.org/10.1016/j.cemconres.2020.106013>.

(9) Zajac, M.; Skibsted, J.; Skocek, J.; Durdzinski, P.; Bullerjahn, F.; Ben Haha, M. Phase assemblage and microstructure of cement paste subjected to enforced, wet carbonation. *Cem. Concr. Res.* **2020**, *130*, 105990. DOI: <https://doi.org/10.1016/j.cemconres.2020.105990>.

(10) Nedunuri, A. S. S. S.; Mohammed, A. y.; Muhammad, S. Carbonation potential of concrete debris fines and its valorisation through mineral carbonation. *Constr. Build. Mater.* **2021**, *310*, 125162. DOI: <https://doi.org/10.1016/j.conbuildmat.2021.125162>.

(11) He, X.; Zeng, J.; Yang, J.; Su, Y.; Wang, Y.; Jin, Z.; Zheng, Z.; Tian, C. Wet grinding carbonation technique: Achieving rapid carbon mineralization of concrete slurry waste under low CO2 flow rate. *Chem. Eng. J.* **2024**, *493*, 152836. DOI: <https://doi.org/10.1016/j.cej.2024.152836>.

(12) Mehdizadeh, H.; Mo, K. H.; Ling, T.-C. CO2-fixing and recovery of high-purity vaterite CaCO3 from recycled concrete fines. *Resour. Conserv. Recycl.* **2023**, *188*, 106695. DOI: <https://doi.org/10.1016/j.resconrec.2022.106695>.

(13) Mao, Y.; He, P.; Drissi, S.; Zhang, J.; Hu, X.; Shi, C. Effect of conditions on wet carbonation products of recycled cement paste powder. *Cem. Concr. Compos.* **2023**, *144*, 105307. DOI: <https://doi.org/10.1016/j.cemconcomp.2023.105307>.

(14) Li, N.; Unluer, C. Enhancement of the wet carbonation of artificial recycled concrete aggregates in seawater. *Cem. Concr. Res.* **2024**, *175*, 107387. DOI: <https://doi.org/10.1016/j.cemconres.2023.107387>.

(15) Jamil, S.; Shi, J.; Idrees, M. Effect of various parameters on carbonation treatment of recycled concrete aggregate using the design of experiment method. *Constr. Build. Mater.* **2023**, *382*, 131339. DOI: <https://doi.org/10.1016/j.conbuildmat.2023.131339>.

(16) Wu, Y.; Mehdizadeh, H.; Mo, K. H.; Ling, T.-C. High-temperature CO2 for accelerating the carbonation of recycled concrete fines. *J. Build. Eng.* **2022**, *52*, 104526. DOI: <https://doi.org/10.1016/j.jobe.2022.104526>.

(17) Kaliyavaradhan, S. K.; Ling, T.-C.; Mo, K. H. CO2 sequestration of fresh concrete slurry waste: Optimization of CO2 uptake and feasible use as a potential cement binder. *J. CO2 Util.* **2020**, *42*, 101330. DOI: <https://doi.org/10.1016/j.jcou.2020.101330>.

(18) Zajac, M.; Song, J.; Skocek, J.; Ben Haha, M.; Skibsted, J. Composite cements with aqueous and semi-dry carbonated recycled concrete pastes. *Constr. Build. Mater.* **2023**, *407*, 133362. DOI: <https://doi.org/10.1016/j.conbuildmat.2023.133362>.

(19) Zajac, M.; Skibsted, J.; Bullerjahn, F.; Skocek, J. Semi-dry carbonation of recycled concrete paste. *J. CO2 Util.* **2022**, *63*, 102111. DOI: <https://doi.org/10.1016/j.jcou.2022.102111>.

(20) Ding, T.; Wong, H.; Qiao, X.; Cheeseman, C. Developing circular concrete: Acid treatment of waste concrete fines. *J. Clean. Prod.* **2022**, *365*, 132615. DOI: <https://doi.org/10.1016/j.jclepro.2022.132615>.

(21) Santha Kumar, G.; Saini, P. K.; Karade, S. R.; Minocha, A. K. Chemico-thermal treatment for quality enhancement of recycled concrete fine aggregates. *J. Mater. Cycles Waste Manage.* **2019**, *21* (5), 1197-1210. DOI: 10.1007/s10163-019-00874-w.

(22) Santha Kumar, G.; Minocha, A. K. Studies on thermo-chemical treatment of recycled concrete fine aggregates for use in concrete. *J. Mater. Cycles Waste Manage.* **2018**, *20* (1), 469-480. DOI: 10.1007/s10163-017-0604-6.

(23) Saravanakumar, P.; Abhiram, K.; Manoj, B. Properties of treated recycled aggregates and its influence on concrete strength characteristics. *Constr. Build. Mater.* **2016**, *111*, 611-617. DOI: <https://doi.org/10.1016/j.conbuildmat.2016.02.064>.

(24) Forero, J. A.; de Brito, J.; Evangelista, L.; Pereira, C. H. F. Mechanical and fracture properties of concrete with recycled concrete aggregates treated with acids and addition of aluminium sulphate. *Constr. Build. Mater.* **2024**, *447*, 137947. DOI: <https://doi.org/10.1016/j.conbuildmat.2024.137947>.

(25) Thaue, W.; Iwanami, M.; Nakayama, K.; Yodsudjai, W. Influence of acetic acid treatment on microstructure of interfacial transition zone and performance of recycled aggregate concrete. *Constr. Build. Mater.* **2024**, *417*, 135355. DOI: <https://doi.org/10.1016/j.conbuildmat.2024.135355>.

(26) Panghal, H.; Kumar, A. Enhancing concrete performance: Surface modification of recycled coarse aggregates for sustainable construction. *Constr. Build. Mater.* **2024**, *411*, 134432. DOI: <https://doi.org/10.1016/j.conbuildmat.2023.134432>.

(27) Verma, A.; Babu, V. S.; Arunachalam, S. Characterization of recycled aggregate by the combined method: Acid soaking and mechanical grinding technique. *Mater. Today: Proc* **2022**, *49*, 230-238. DOI: <https://doi.org/10.1016/j.matpr.2021.01.842>.

(28) Andal N, M.; R, T.; A, B. Strength enhancement of recycled fine aggregate beam through chemical treatment - A waste to wealth Approach. *J. Mater. Res. Technol.* **2024**, *30*, 7340-7351. DOI: <https://doi.org/10.1016/j.jmrt.2024.05.069>.

(29) Tanta, A.; Kanoungo, A.; Singh, S.; Kanoungo, S. The effects of surface treatment methods on properties of recycled concrete aggregates. *Mater. Today: Proc* **2022**, *50*, 1848-1852. DOI: <https://doi.org/10.1016/j.matpr.2021.09.223>.

(30) Al-Bayati, H. K. A.; Das, P. K.; Tighe, S. L.; Baaj, H. Evaluation of various treatment methods for enhancing the physical and morphological properties of coarse recycled concrete aggregate. *Constr. Build. Mater.* **2016**, *112*, 284-298. DOI: <https://doi.org/10.1016/j.conbuildmat.2016.02.176>.

(31) Dilbas, H.; Çakır, Ö.; Atiş, C. D. Experimental investigation on properties of recycled aggregate concrete with optimized Ball Milling Method. *Constr. Build. Mater.* **2019**, *212*, 716-726. DOI: <https://doi.org/10.1016/j.conbuildmat.2019.04.007>.

(32) He, X.; Ma, Q.; Su, Y.; Zheng, Z.; Tan, H.; Peng, K.; Zhao, R. Humid hardened concrete waste treated by multiple wet-grinding and its reuse in concrete. *Constr. Build. Mater.* **2022**, *350*, 128485. DOI: <https://doi.org/10.1016/j.conbuildmat.2022.128485>.

(33) Prajapati, R.; Gettu, R.; Singh, S. Thermomechanical beneficiation of recycled concrete aggregates (RCA). *Constr. Build. Mater.* **2021**, *310*, 125200. DOI: <https://doi.org/10.1016/j.conbuildmat.2021.125200>.

(34) Gupta, S.; Agrawal, H.; Chaudhary, S. Thermo-mechanical treatment as an upcycling strategy for mixed recycled aggregate. *Constr. Build. Mater.* **2023**, *398*, 132471. DOI: <https://doi.org/10.1016/j.conbuildmat.2023.132471>.

(35) Yoon, H.-S.; Seo, E.-A.; Kim, D.-G.; Yang, K.-H. Efficiency of dry calcination and trituration treatments for removing cement pastes attached to recycled coarse aggregates. *Constr. Build. Mater.* **2021**, *312*, 125412. DOI: <https://doi.org/10.1016/j.conbuildmat.2021.125412>.

(36) Bui, N. K.; Satomi, T.; Takahashi, H. Mechanical properties of concrete containing 100% treated coarse recycled concrete aggregate. *Constr. Build. Mater.* **2018**, *163*, 496-507. DOI: <https://doi.org/10.1016/j.conbuildmat.2017.12.131>.

(37) Alqarni, A. S.; Abbas, H.; Al-Shwikh, K. M.; Al-Salloum, Y. A. Treatment of recycled concrete aggregate to enhance concrete performance. *Constr. Build. Mater.* **2021**, *307*, 124960. DOI: <https://doi.org/10.1016/j.conbuildmat.2021.124960>.

(38) Chen, W.-Z.; Jiao, C.-J.; Zhang, X.-C.; Yang, Y.; Chen, X.-F. Study on the microstructure of recycled aggregate concrete strengthened by the nano-SiO2 soaking method. *Structures* **2023**, *58*, 105388. DOI: <https://doi.org/10.1016/j.istruc.2023.105388>.

(39) Siletani, A. H.; Asayesh, S.; Shirzadi Javid, A. A.; Habibnejad Korayem, A.; Ghanbari, M. A. Influence of coating recycled aggregate surface with different pozzolanic slurries on mechanical performance, durability, and micro-structure properties of recycled aggregate concrete. *J. Build. Eng.* **2024**, *83*, 108457. DOI: <https://doi.org/10.1016/j.jobe.2024.108457>.

(40) Zhou, Y.; Zhuang, J.; Xu, W.; Lin, W.; Xing, F.; Hu, R. Study on mechanical performance and mesoscopic simulation of nano-SiO2 modified recycled aggregate concrete. *Constr. Build. Mater.* **2024**, *425*, 136053. DOI: <https://doi.org/10.1016/j.conbuildmat.2024.136053>.

(41) Zhao, W.; Liu, J.; Guo, H.; Li, L. Effect of nano-SiO2 modified recycled coarse aggregate on the mechanical properties of recycled concrete. *Constr. Build. Mater.* **2023**, *395*, 132319. DOI: <https://doi.org/10.1016/j.conbuildmat.2023.132319>.

(42) Liu, X.; Xie, X.; Liu, R.; Lyu, K.; Zuo, J.; Li, S.; Liu, L.; Shah, S. P. Research on the durability of nano-SiO2 and sodium silicate co-modified recycled coarse aggregate (RCA) concrete. *Constr. Build. Mater.* **2023**, *378*, 131185. DOI: <https://doi.org/10.1016/j.conbuildmat.2023.131185>.

(43) Santos, W. F.; Quattrone, M.; John, V. M.; Angulo, S. C. Roughness, wettability and water absorption of water repellent treated recycled aggregates. *Constr. Build. Mater.* **2017**, *146*, 502-513. DOI: <https://doi.org/10.1016/j.conbuildmat.2017.04.012>.

(44) Geng, W.; Li, C.; Zeng, D.; Chen, J.; Wang, H.; Liu, Z.; Liu, L. Effect of epoxy resin surface-modified techniques on recycled coarse aggregate and recycled aggregate concrete. *J. Build. Eng.* **2023**, *76*, 107081. DOI: <https://doi.org/10.1016/j.jobe.2023.107081>.

(45) Zou, D.; Wang, Z.; Shen, M.; Liu, T.; Zhou, A. Improvement in freeze-thaw durability of recycled aggregate permeable concrete with silane modification. *Constr. Build. Mater.* **2021**, *268*, 121097. DOI: <https://doi.org/10.1016/j.conbuildmat.2020.121097>.

(46) Liu, T.; Wang, Z.; Zou, D.; Zhou, A.; Du, J. Strength enhancement of recycled aggregate pervious concrete using a cement paste redistribution method. *Cem. Concr. Res.* **2019**, *122*, 72-82. DOI: <https://doi.org/10.1016/j.cemconres.2019.05.004>.

(47) Sua-iam, G.; Makul, N. Self-compacting concrete produced with recycled concrete aggregate coated by a polymer-based agent: A case study. *Case Stud. Constr. Mater.* **2023**, *19*, e02351. DOI: <https://doi.org/10.1016/j.cscm.2023.e02351>.

(48) Velardo, P.; Sáez del Bosque, I. F.; Matías, A.; Sánchez de Rojas, M. I.; Medina, C. Properties of concretes bearing mixed recycled aggregate with polymer-modified surfaces. *J. Build. Eng.* **2021**, *38*, 102211. DOI: <https://doi.org/10.1016/j.jobe.2021.102211>.

(49) Velardo, P.; Sáez del Bosque, I. F.; Sánchez de Rojas, M. I.; De Belie, N.; Medina, C. Durability of concrete bearing polymer-treated mixed recycled aggregate. *Constr. Build. Mater.* **2022**, *315*, 125781. DOI: <https://doi.org/10.1016/j.conbuildmat.2021.125781>.

(50) Zhang, R.; Xie, D.; Wu, K.; Wang, J. Optimization of sodium alginate aided bio-deposition treatment of recycled aggregates and its application in concrete. *Cem. Concr. Compos.* **2023**, *139*, 105031. DOI: <https://doi.org/10.1016/j.cemconcomp.2023.105031>.

(51) Wu, C.-R.; Zhu, Y.-G.; Zhang, X.-T.; Kou, S.-C. Improving the properties of recycled concrete aggregate with bio-deposition approach. *Cem. Concr. Compos.* **2018**, *94*, 248-254. DOI: <https://doi.org/10.1016/j.cemconcomp.2018.09.012>.

(52) Wang, J.; Vandevyvere, B.; Vanhessche, S.; Schoon, J.; Boon, N.; De Belie, N. Microbial carbonate precipitation for the improvement of quality of recycled aggregates. *J. Cleaner Prod.* **2017**, *156*, 355-366. DOI: <https://doi.org/10.1016/j.jclepro.2017.04.051>.

(53) Yang, Y.; Tian, D.; Gao, P.; Zhan, B.; Yu, Q.; Wang, J.; Wang, A.; Ni, M.; Zhao, P.; Zhang, Y.; et al. Study on the effect and mechanism of microwave excitation on the activity of recycled powder of waste concrete. *Constr. Build. Mater.* **2024**, *429*, 136410. DOI: <https://doi.org/10.1016/j.conbuildmat.2024.136410>.

(54) Zhang, S.; Tan, H.; Yang, L.; Luo, S. Impact of microwave radiation on recycled concrete powder in cement-based materials: Structure, hydration activity and mechanism. *J. Build. Eng.* **2024**, *86*, 108864. DOI: <https://doi.org/10.1016/j.jobe.2024.108864>.

(55) association, C. m. *Material Quality Control in Cement Manufacturing*. 2022. <https://www.cmaindia.org/material-quality-control-cement> (accessed.

(56) ISO. *Environmental management: life cycle assessment; Principles and Framework*; ISO, 2006.

(57) Standard, I. *Environmental management-Life cycle assessment-Requirements and guidelines*; ISO, 2006.

(58) Gálvez-Martos, J.-L.; Chaliulina, R.; Elhoweris, A.; Mwanda, J.; Hakki, A.; Al-horr, Y. Techno-economic assessment of calcium sulfoaluminate clinker production using elemental sulfur as raw material. *J. Clean. Prod.* **2021**, *301*, 126888. DOI: <https://doi.org/10.1016/j.jclepro.2021.126888>.

(59) Akintola, J. T.; Fadayini, O.; Madu, C.; Oshin, T.; Obisanya, A.; Ajiboye, G.; Ipaye, T.; Rabiu, T.; Ajayi, S. J.; Kingsley, N. A. Energy and Economic Comparison of Different Fuels in Cement Production. In *Cement Industry - Optimization, Characterization and Sustainable Application*, Saleh, H. M. Ed.; IntechOpen, 2021.

(60) "Smith, I. *Co-utilisation of coal and other fuels in cement kilns*; CCC-71;Other: ISBN 92-9029-386-1; TRN: 001100299; IEA Clean Coal Centre, London (United Kingdom); IEA Clean Coal Centre, London (United Kingdom), United Kingdom, 2003. DOI: <https://doi.org/> Other: ISBN 92-9029-386-1; TRN: 001100299 CLA.

(61) Hasanbeigi, A., Bhadbhade, N. *Emissions Impacts of Alternative Fuels Combustion in the Cement Industry*; Global Efficiency Intelligence. Florida, United States., 2023.

(62) Akhtar, S. S.; Ervin, E.; Raza, S.; Abbas, T. From Coal to Natural Gas: Its Impact on Kiln Production, Clinker Quality, and Emissions. *IEEE Trans. Ind. Appl.* **2016**, *52* (2), 1913-1924. DOI: 10.1109/TIA.2015.2504554.

(63) Xi, F.; Davis, S. J.; Ciais, P.; Crawford-Brown, D.; Guan, D.; Pade, C.; Shi, T.; Syddall, M.; Lv, J.; Ji, L.; et al. Substantial global carbon uptake by cement carbonation. *Nature Geoscience* **2016**, *9* (12), 880-883. DOI: 10.1038/ngeo2840.

(64) Jungclaus, M. A.; Williams, S. L.; Arehart, J. H.; Srubar, W. V. Whole-life carbon emissions of concrete mixtures considering maximum CO2 sequestration via carbonation. *Resour. Conserv. Recycl.* **2024**, *206*, 107605. DOI: <https://doi.org/10.1016/j.resconrec.2024.107605>.

(65) Cao, Z.; Myers, R. J.; Lupton, R. C.; Duan, H.; Sacchi, R.; Zhou, N.; Reed Miller, T.; Cullen, J. M.; Ge, Q.; Liu, G. The sponge effect and carbon emission mitigation potentials of the global cement cycle. *Nat. Commun.* **2020**, *11* (1), 3777. DOI: 10.1038/s41467-020-17583-w.

(66) Nakic, D. Environmental evaluation of concrete with sewage sludge ash based on LCA. *Sustainable Production and Consumption* **2018**, *16*, 193-201. DOI: <https://doi.org/10.1016/j.spc.2018.08.003>.

(67) Hossain, M. U.; Poon, C. S.; Lo, I. M. C.; Cheng, J. C. P. Comparative LCA on using waste materials in the cement industry: A Hong Kong case study. *Resour. Conserv. Recycl.* **2017**, *120*, 199-208. DOI: <https://doi.org/10.1016/j.resconrec.2016.12.012>.

(68) *Run-of-river hydropower*. UN Climate Technology Centre & Network, <https://www.ctc-n.org/technologies/run-river-hydropower> (accessed.

(69) Zhang, Z.; Mowbray, B. A. W.; Parkyn, C. T. E.; Waizenegger, C.; Williams, A. S. R.; Lees, E. W.; Ren, S.; Kim, Y.; Jansonius, R. P.; Berlinguette, C. P. Cement clinker precursor production in an electrolyser. *Energy & Environmental Science* **2022**, *15* (12), 5129-5136, 10.1039/D2EE02349K. DOI: 10.1039/D2EE02349K.

(70) Ellis, L. D.; Badel, A. F.; Chiang, M. L.; Park, R. J.-Y.; Chiang, Y.-M. Toward electrochemical synthesis of cement: An electrolyzer-based process for decarbonating CaCO3 while producing useful gas streams. *PNAS* **2020**, *117* (23), 12584-12591. DOI: doi:10.1073/pnas.1821673116.

(71) *Heat Values of Various Fuels*. World Nuclear Association, 2020. <https://world-nuclear.org/information-library/facts-and-figures/heat-values-of-various-fuels> (accessed.

(72) Daniel Münter, A. L. *Analysis of the greenhouse gas intensities of LNG imports to Germany*; Institut für Energie- und Umweltforschung, 2023.

(73) *Specific Carbon Dioxide Emissions of Various Fuels*. Volker-quaschining, 2022. <https://www.volker-quaschning.de/datserv/CO2-spez/index_e.php> (accessed.

(74) Hossain, M. U.; Poon, C. S.; Lo, I. M. C.; Cheng, J. C. P. Comparative environmental evaluation of aggregate production from recycled waste materials and virgin sources by LCA. *Resour. Conserv. Recycl.* **2016**, *109*, 67-77. DOI: <https://doi.org/10.1016/j.resconrec.2016.02.009>.

(75) Zhang, M.; Liu, X.; Kong, L. Evaluation of carbon and economic benefits of producing recycled aggregates from construction and demolition waste. *J. Clean. Prod.* **2023**, *425*, 138946. DOI: <https://doi.org/10.1016/j.jclepro.2023.138946>.

(76) Laveglia, A.; Sambataro, L.; Ukrainczyk, N.; De Belie, N.; Koenders, E. Hydrated lime life-cycle assessment: Current and future scenarios in four EU countries. *J. Clean. Prod.* **2022**, *369*, 133224. DOI: <https://doi.org/10.1016/j.jclepro.2022.133224>.

(77) Zhang, S.; Yuan, Q.; Ni, J.; Zheng, K.; Xu, Y.; Zhang, J. CO2 utilization and sequestration in ready-mix concrete—A review. *Sci. Total Environ.* **2024**, *907*, 168025. DOI: <https://doi.org/10.1016/j.scitotenv.2023.168025>.

(78) Zhang, J.; Cheng, J. C. P.; Lo, I. M. C. Life cycle carbon footprint measurement of Portland cement and ready mix concrete for a city with local scarcity of resources like Hong Kong. *The International Journal of Life Cycle Assessment* **2014**, *19* (4), 745-757. DOI: 10.1007/s11367-013-0689-7.

(79) Wang, J.; Wei, J.; Liu, Z.; Huang, C.; Du, X. Life cycle assessment of building demolition waste based on building information modeling. *Resour. Conserv. Recycl.* **2022**, *178*, 106095. DOI: <https://doi.org/10.1016/j.resconrec.2021.106095>.

(80) US, D. o. E. *U.S. Hydropower Market Report 2023 Edition*. 2025. <https://www.energy.gov/sites/default/files/2023-09/U.S.%20Hydropower%20Market%20Report%202023%20Edition.pdf> (accessed.

(81) Klein, S. J. W.; Fox, E. L. B. A review of small hydropower performance and cost. *Renewable and Sustainable Energy Reviews* **2022**, *169*, 112898. DOI: <https://doi.org/10.1016/j.rser.2022.112898>.

(82) Amir Raza, M.; Karim, A.; Aman, M. M.; Ahmad Al-Khasawneh, M.; Faheem, M. Global progress towards the Coal: Tracking coal reserves, coal prices, electricity from coal, carbon emissions and coal phase-out. *Gondwana Research* **2025**, *139*, 43-72. DOI: <https://doi.org/10.1016/j.gr.2024.11.007>.

(83) *Natural gas prices*. GlobalPetrolPrices, 2024. <https://www.globalpetrolprices.com/natural_gas_prices/> (accessed.

(84) Mokrzycki, E.; Uliasz- Bocheńczyk, A. Alternative fuels for the cement industry. *Appl. Energy* **2003**, *74* (1), 95-100. DOI: <https://doi.org/10.1016/S0306-2619(02)00135-6>.

(85) Fennell, P. S.; Davis, S. J.; Mohammed, A. Decarbonizing cement production. *Joule* **2021**, *5* (6), 1305-1311. DOI: 10.1016/j.joule.2021.04.011 (acccessed 2025/07/11).

(86) Guikema, A. *An Overview of Global Carbon Pricing in 2024*. Sustainable Markets, 2024. <https://www.sustainable-markets.com/2024/07/16/an-overview-of-global-carbon-pricing-in-2024/> (accessed.

(87) Council, N. P. U. *Visualized: The Price of Carbon Around the World*. 2024. <https://www.motive-power.com/visualized-the-price-of-carbon-around-the-world/> (accessed.

(88) ENERGY, T. S. *Cement costs and energy economics?* 2024. <https://thundersaidenergy.com/downloads/cement-costs-and-energy-economics/> (accessed.

(89) Statista. *Cement prices in the United States from 2010 to 2024*. 2025. <https://www.statista.com/statistics/219339/us-prices-of-cement/> (accessed.

(90) Li, J.; Zuo, J.; Wang, G.; He, G.; Tam, V. W. Y. Stakeholders’ willingness to pay for the new construction and demolition waste landfill charge scheme in Shenzhen: A contingent valuation approach. *Sustainable Cities and Society* **2020**, *52*, 101663. DOI: <https://doi.org/10.1016/j.scs.2019.101663>.

(91) Caro, D.; Lodato, C.; Damgaard, A.; Cristóbal, J.; Foster, G.; Flachenecker, F.; Tonini, D. Environmental and socio-economic effects of construction and demolition waste recycling in the European Union. *Sci. Total Environ.* **2024**, *908*, 168295. DOI: <https://doi.org/10.1016/j.scitotenv.2023.168295>.

(92) Ehinger, B. *How Much Is It to Go to the Dump: Understanding Disposal Costs*. WASTE REMOVAL USA, 2024. <https://wasteremovalusa.com/blog/how-much-is-it-to-go-to-the-dump/> (accessed.
